# ZBTB7B is a permissive regulator of hepatocellular carcinoma initiation by repressing c-Jun expression and function

**DOI:** 10.1038/s41419-024-06441-y

**Published:** 2024-01-15

**Authors:** Yue Zhu, Qinqin Wang, Xinyu Xie, Cuihong Ma, Yuemei Qiao, Yu Zhang, Yanjun Wu, Yuan Gao, Jing Jiang, Xin Liu, Jianfeng Chen, Chen Li, Gaoxiang Ge

**Affiliations:** 1 State Key Laboratory of Cell Biology, Center for Excellence in Molecular Cell Science, Shanghai Institute of Biochemistry and Cell Biology, University of Chinese Academy of Sciences, Chinese Academy of Sciences, Shanghai, 200031 China; 2 Center for Single-Cell Omics, School of Public Health, Shanghai Jiao Tong University School of Medicine, Shanghai, 200025 China; 3 State Key Laboratory of Molecular Biology, Center for Excellence in Molecular Cell Science, Shanghai Institute of Biochemistry and Cell Biology, Chinese Academy of Sciences, Shanghai, 200031 China; 4 Genome Tagging Project (GTP) Center, Center for Excellence in Molecular Cell Science, Shanghai Institute of Biochemistry and Cell Biology, Chinese Academy of Sciences, Shanghai, 200031 China; 5 Key Laboratory of Systems Health Science of Zhejiang Province, School of Life Science, Hangzhou Institute for Advanced Study, University of Chinese Academy of Sciences, Hangzhou, 310024 China

**Keywords:** Liver cancer, Liver cancer

## Abstract

Hepatocarcinogenesis is a multi-step process. However, the regulators of hepatocellular carcinoma (HCC) initiation are understudied. Adult liver-specific gene expression was globally downregulated in HCC. We hypothesize that adult liver-specific genes, especially adult liver-enriched transcription factors may exert tumor-suppressive functions in HCC. In this study, we identify ZBTB7B, an adult liver-enriched transcription factor as a permissive regulator of HCC initiation. ZBTB7B is highly expressed in hepatocytes in adult livers, compared to fetal livers. To evaluate the functions of ZBTB7B in hepatocarcinogenesis, we performed hepatocyte-specific ZBTB7B knockout in hydrodynamic oncogene transfer-induced mouse liver cancer models. Hepatocyte-specific knockout of ZBTB7B promotes activated Akt and N-Ras-induced HCC development. Moreover, ZBTB7B deficiency sensitizes hepatocytes to a single oncogene Akt-induced oncogenic transformation and HCC initiation, which is otherwise incompetent in inducing HCC. ZBTB7B deficiency accelerates HCC initiation by down-regulating adult liver-specific gene expression and priming livers to a fetal-like state. The molecular mechanism underlying ZBTB7B functions in hepatocytes was investigated by integrated transcriptomic, phosphoproteomic, and chromatin immunoprecipitation-sequencing analyses. Integrative multi-omics analyses identify c-Jun as the core signaling node in ZBTB7B-deficient liver cancer initiation. c-Jun is a direct target of ZBTB7B essential to accelerated liver cancer initiation in ZBTB7B-deficient livers. Knockdown of c-Jun expression or dominant negative c-Jun expression delays HCC development in ZBTB7B-deficient livers. In addition, ZBTB7B competes with c-Jun for chromatin binding. Ectopic ZBTB7B expression attenuates the tumor-promoting functions of c-Jun. Expression of ZBTB7B signature, composed of 140 genes co-regulated by ZBTB7B and c-Jun, is significantly downregulated in early-stage HCCs compared to adjacent normal tissues, correlates to liver-specific gene expression, and is associated with good prognosis in human HCC. Thus, ZBTB7B functions as a permissive regulator of HCC initiation by directly regulating c-Jun expression and function.

## Introduction

Hepatocellular carcinoma (HCC), the major type of primary liver cancer, is unfortunately often diagnosed when the disease is already at advanced stages. It is therefore of special importance to fully understand how malignant transformation occurs in early-stage preneoplastic lesions. Hepatocarcinogenesis is a multi-step process progressing from regenerative nodules to early HCC (ref. [1, 2]). Genetic alterations driving HCC development, e.g., *TERT* promoter, *TP53*, are rarely found in preneoplastic lesions of HCC (ref. [3–5]), suggesting deregulated expression or functions of signaling molecules contribute to HCC initiation (ref. [2–6]). Intriguingly, transcriptomic and proteomic analyses of HCC reveal that genes downregulated in HCC are mostly those specifically expressed in differentiated hepatocytes (ref. [7, 8]). Global downregulation of the adult liver-specific proteins in HCC may be attributed to impaired functions of liver-specific transcription factors that dictate hepatocyte differentiation and regulate liver-specific functions. We hypothesize that deregulated functions of these transcription factors may have pivotal roles in the initiation of HCC.

Transcription factor zinc finger and BTB domain-containing 7b (ZBTB7B, also known as Th-POK or cKrox) is a critical regulator of cell fate determination in many biological processes. ZBTB7B is a key regulator in T cell lineage commitment (ref. [9–11]). Loss of ZBTB7B expression or impaired ZBTB7B function disrupts the development of CD4 T cells (ref. [12]). ZBTB7B expression is temporally induced during BMP4-induced reprogramming of mouse epiblast stem cells to naive pluripotency (ref. [13]). ZBTB7B facilitates the opening of naive pluripotent chromatin loci and the primed-to-naive transition (ref. [13]). ZBTB7B is a potent driver of brown fat development and cold-induced beige fat formation that activates thermogenic gene expression in adipocytes (ref. [14]). Besides cell fate specification, ZBTB7B is also involved in metabolic regulation. ZBTB7B regulates the expression of insulin receptor substrate-1 (IRS-1) (ref. [15]). ZBTB7B deficiency impairs insulin-induced Akt-mTOR-SREBP pathway and lipid biosynthesis in the mammary alveolar cells at lactation (ref. [15]).

In this study, we report that ZBTB7B is highly expressed in adult hepatocytes and is a negative regulator of HCC initiation. ZBTB7B deficiency primes hepatocytes to a fetal state, sensitizes hepatocytes to oncogenic transformation, and accelerates HCC initiation. ZBTB7B exerts tumor-suppressive functions by directly regulating c-Jun expression and function.

## Results

### ZBTB7B is an adult liver-enriched transcription factor

Adult liver-specific genes (ref. [16]) are globally downregulated in human HCC (Fig. S1A) (ref. [7, 8]). Combined overexpression of HA tagged-activated Akt (Myr-Akt-HA) and N-Ras (N-Ras V12) in the liver via hydrodynamic gene transfer induced rapid tumor development (ref. [17]). Similar to human HCC, adult liver-specific gene expression was substantially downregulated in Akt/N-Ras-induced tumors (Fig. S1B). We hypothesize that adult liver-specific genes, especially adult liver-enriched transcription factors may exert tumor-suppressive functions in HCC. Livers undergo postnatal maturation to establish functional organs. Clustering analysis of fetal and adult liver gene expression identified six hepatic gene clusters (Fig. S1C). Clusters 1, 2, 3, 5, and 6 represented genes that were highly expressed in stages of embryonic development, but were expressed at low levels in adult livers (Fig. S1C), including oncofetal genes *Afp*, *Gpc3*, *Sall4* (cluster 2), *H19* (Cluster 3) and *Igf2* (Cluster 5), which are re-expressed in liver cancer (ref. [18–22]). Cluster 4 represented genes that were expressed at low levels in fetal livers, but high levels in adult livers (Fig. S1C). Top adult liver-specific transcription factors in cluster 4 included *Nr1h3*/*Lxra* and *Rxra*, transcription factors critical for adult liver functions (ref. [23]) (Fig. S1D). Cluster 4 also included *Esr1* and *Smad7*, which were previously reported to be inhibitory to HCC development (ref. [24, 25]) (Fig. S1D). Interestingly, *Zbtb7b*, a transcription factor regulating cell fate determination, shared similar expression kinetics to *Nr1h3*/*Lxra*, *Rxra*, *Esr1*, and *Smad7* (Fig. S1D, E). Hepatic ZBTB7B mRNA and protein levels were significantly increased during the postnatal maturation process (Fig. S1F–H). Expression of ZBTB7B was also increased in adult human liver, compared to human fetal liver (Fig. S1I).

**Fig. 1:**
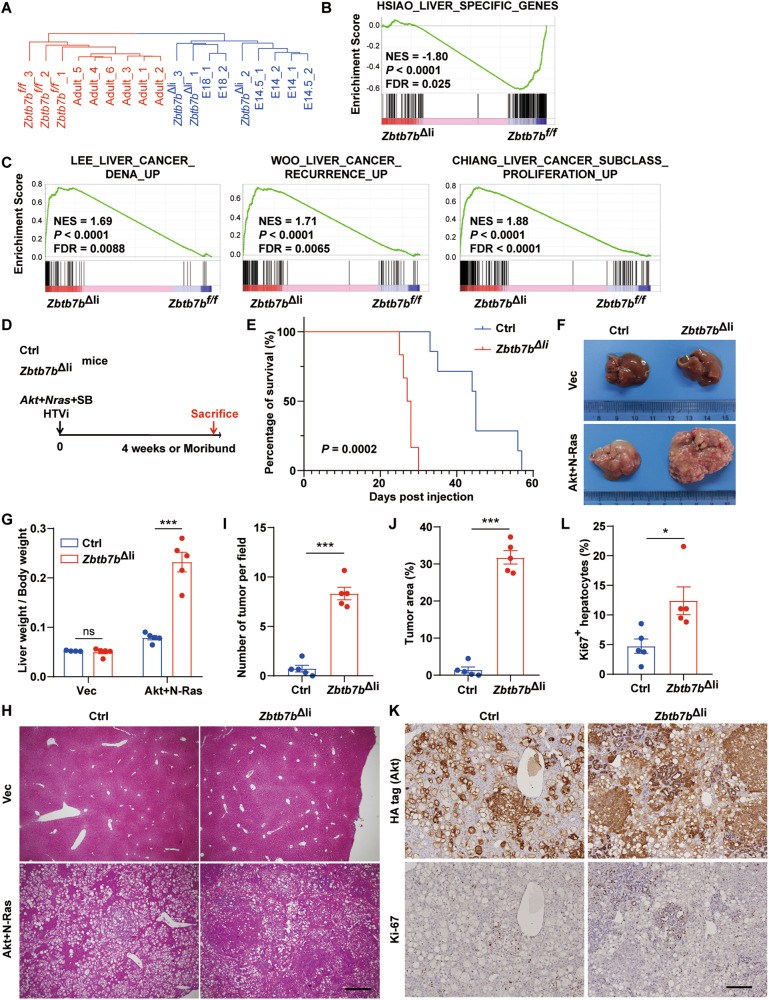
ZBTB7B deficiency accelerates Akt/N-Ras-induced liver cancer. **A** Phylogenetic analysis of gene expression in the *Zbtb7b*^*f/f*^ and *Alb*-*Cre*, *Zbtb7b*^*f/f*^ (*Zbtb7b*^Δli^) adult livers, and published gene expression profiles of adult and fetal livers. **B** GSEA comparison of liver-specific gene expression between the *Zbtb7b*^*f/f*^ and *Zbtb7b*^Δli^ livers. **C** GSEA comparison of liver cancer gene signatures between the *Zbtb7b*^*f/f*^ and *Zbtb7b*^Δli^ livers. **D** Study design. Hydrodynamic tail vein injection (HTVi) of Akt and N-Ras to induce tumor development in control (*Alb*-*Cre* or *Zbtb7b*^*f/f*^, Ctrl) or *Zbtb7b*^Δli^ mice. **E** Kaplan–Meier survival analysis. Control (*n* = 6), *Zbtb7b*^Δli^ (*n* = 7). **F** Gross liver images of control and *Zbtb7b*^Δli^ mice 4 weeks after injection with vector (Vec) or Akt/N-Ras. **G** Liver/body weight ratio of control and *Zbtb7b*^Δli^ mice 4 weeks after injection with vector or Akt/N-Ras. *n* = 5. **H** H&E staining of liver sections of control and *Zbtb7b*^Δli^ mice 4 weeks after injection with vector or Akt/N-Ras. Magnification: ×4. Scale bar: 500 μm. **I**, **J** Numbers of tumors (**I**) and percentage of tumor area (**J**) in the livers of control and *Zbtb7b*^Δli^ mice 4 weeks after Akt/N-Ras injection. *n* = 5. **K** Immunohistochemistry of HA-tag and Ki67 on liver sections of control and *Zbtb7b*^Δli^ mice 4 weeks after Akt/N-Ras injection. Magnification: ×10. Scale bar: 200 μm. **L** Percentage of Ki67^+^ hepatocytes in the livers of control and *Zbtb7b*^Δli^ mice 4 weeks after Akt/N-Ras injection. *n* = 5. Data are presented as mean ± SEM. Statistical analyses were performed with the Log-Rank test (**E**), two-way ANOVA followed by Šídák’s multiple comparison tests (**G**) or two-tailed unpaired student’s *t* test (**I**, **J**, **L**). *P < 0.05, ***P < 0.001. ns: not significant.

ZBTB7B is a critical regulator of cell fate determination in many biological processes (ref. [9, 13, 14]). It is yet unknown whether ZBTB7B regulates hepatocyte differentiation, liver functions, and liver cancer development. Immunostaining on liver sections revealed that ZBTB7B was mainly expressed in hepatocytes in the liver, and ~90% of hepatocytes expressed ZBTB7B (Fig. S2). To investigate the functions of hepatic ZBTB7B, *Zbtb7b*^*f/f*^ mice were crossed with *Alb*-*Cre* mice to specifically delete *Zbtb7b* in the hepatocytes (*Zbtb7b*^Δli^) (Fig. S2 and S3A). Despite no overt developmental abnormality in the hepatocyte *Zbtb7b*-ablated livers (Fig. S3A–E), phylogenetic analysis of liver transcriptomes revealed that *Zbtb7b*^Δli^ livers had gene expression pattern resembling the fetal livers (Fig. 1A). Notably, expression of the adult liver-specific genes was significantly downregulated in the *Zbtb7b*^Δli^ livers (Fig. 1B). Liver cancer gene signatures were enriched (Fig. 1C) and significantly more Ki67^+^ proliferating hepatocytes were present in the *Zbtb7b*^Δli^ livers (Fig. S3F, G). Upon activated Akt/N-Ras expression, ZBTB7B expression was significantly downregulated (Fig. S3H). As deficiency of ZBTB7B, an adult liver-enriched cell fate regulator, in hepatocytes primed the livers to a fetal-like state and its expression was downregulated in the tumors, we next sought to investigate whether hepatocyte ZBTB7B would function as a tumor suppressor in regulating HCC development.

### ZBTB7B deficiency accelerates activated Akt/N-Ras-induced hepatocarcinogenesis

Combined overexpression of activated Akt and N-Ras induced tumor development in the livers of *Alb*-*Cre* or *Zbtb7b*^*f/f*^ (control, Ctrl) mice with median survival of 45.0 days (Fig. 1D, E) (ref. [17]). Ablation of *Zbtb7b* in hepatocytes significantly accelerated Akt/N-Ras-induced liver cancer development with median survival of 27.5 days (Fig. 1D, E). At 4 weeks after Akt/N-Ras oncogene transfer, *Zbtb7b*^Δli^ mice had significantly more tumors with a much higher liver/body weight ratio than the control mice (Fig. 1F, G). Akt/N-Ras induced multifocal tumors in the liver (Fig. 1H). Significantly more tumors developed in the *Zbtb7b*^Δli^ mice with larger tumor size, compared to the control mice (Fig. 1H–J). Immunostaining of the HA-tag showed more exogenous Akt-expressing cells in the *Zbtb7b*^Δli^ livers (Fig. 1K). *Zbtb7b*^Δli^ mice had significantly more Ki67^+^ proliferative hepatocytes (Fig. 1K, L). To further investigate whether ZBTB7B suppresses liver cancer development, ZBTB7B was expressed along with Akt/N-Ras oncogenes in the wild-type mice (Fig. S4A). Ectopic ZBTB7B expression alleviated tumor burden with reduced tumor numbers, tumor area, and proliferative hepatocyte numbers (Fig. S4). These data collectively suggested that ZBTB7B exerts tumor-suppressive functions in Akt/N-Ras oncogene-induced liver cancer development.

### ZBTB7B deficiency accelerates liver cancer initiation and is permissive to single oncogene-induced hepatocarcinogenesis

**Fig. 2:**
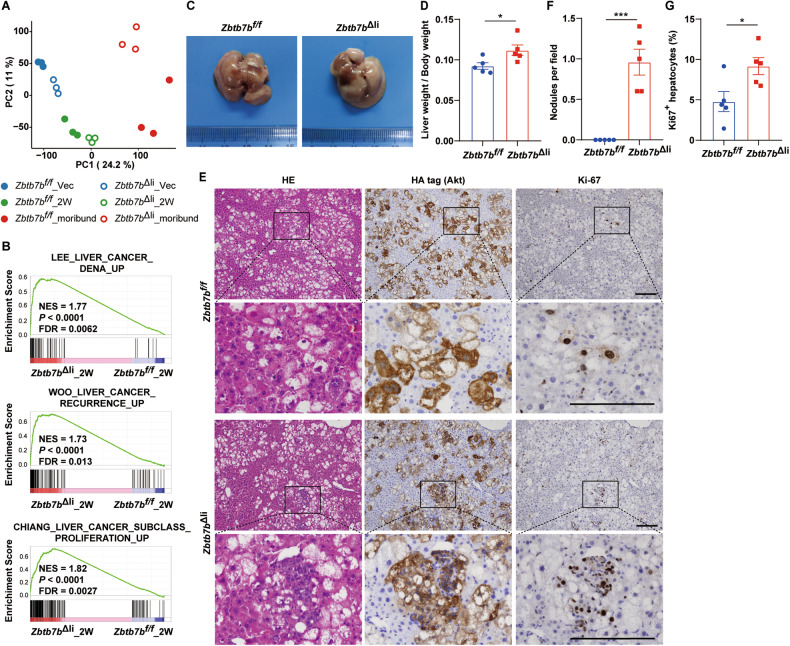
ZBTB7B deficiency accelerates liver cancer initiation. **A** Principal component analysis of gene expression in the livers of *Zbtb7b*^*f/f*^ and *Alb*-*Cre*, *Zbtb7b*^*f/f*^ (*Zbtb7b*^Δli^) mice injected with vector or Akt/N-Ras. **B** GSEA comparison of liver cancer gene signatures between *Zbtb7b*^*f/f*^ and *Zbtb7b*^Δli^ livers 2 weeks after Akt/N-Ras injection. **C** Gross liver images of *Zbtb7b*^*f/f*^ and *Zbtb7b*^Δli^ mice 2 weeks after Akt/N-Ras injection. **D** Liver/body weight ratio of *Zbtb7b*^*f/f*^ and *Zbtb7b*^Δli^ mice 2 weeks after Akt/N-Ras injection. *n* = 5. **E** H&E staining and immunohistochemistry of HA-tag and Ki67 on the liver sections of *Zbtb7b*^*f/f*^ and *Zbtb7b*^Δli^ mice 2 weeks after Akt/N-Ras injection. Magnifications: ×10 and ×40. Scale bars: 200 μm. **F**, **G** Numbers of tumor nodules (**F**) and percentage of Ki67^+^ hepatocytes (**G**) in the livers of *Zbtb7b*^*f/f*^ and *Zbtb7b*^Δli^ mice 2 weeks after Akt/N-Ras injection. *n* = 5. Data are presented as mean ± SEM. Statistical analyses were performed with two-tailed unpaired student’s *t* test. *P < 0.05, ***P < 0.001.

Combined Akt/N-Ras oncogene expression drives stepwise hepatocarcinogenesis (ref. [17]). Consistent with histological changes, principal component analysis of gene expression in the livers transduced with Akt/N-Ras oncogenes showed stepwise global gene expression changes (Fig. 2A). The changes on gene expression in the *Zbtb7b*^Δli^ livers preceded that in the *Zbtb7b*^*f/f*^ livers (Fig. 2A), suggesting liver cancer initiation occurred at earlier time point in the *Zbtb7b*^Δli^ livers than the *Zbtb7b*^*f/f*^ livers. At 2 weeks after Akt/N-Ras oncogene transfer, liver cancer gene signatures were enriched in the *Zbtb7b*^Δli^ livers compared to the *Zbtb7b*^*f/f*^ livers (Fig. 2B). *Zbtb7b*^Δli^ livers appeared paler than the *Zbtb7b*^*f/f*^ livers with higher liver/body weight ratio (Fig. 2C, D). Despite no tumor nodule being evident in the *Zbtb7b*^*f/f*^ livers at this time point (Fig. 2E, F), multiple HA-tag-positive, highly proliferative tumor nodules developed in the *Zbtb7b*^Δli^ livers (Fig. 2E–G).

**Fig. 3:**
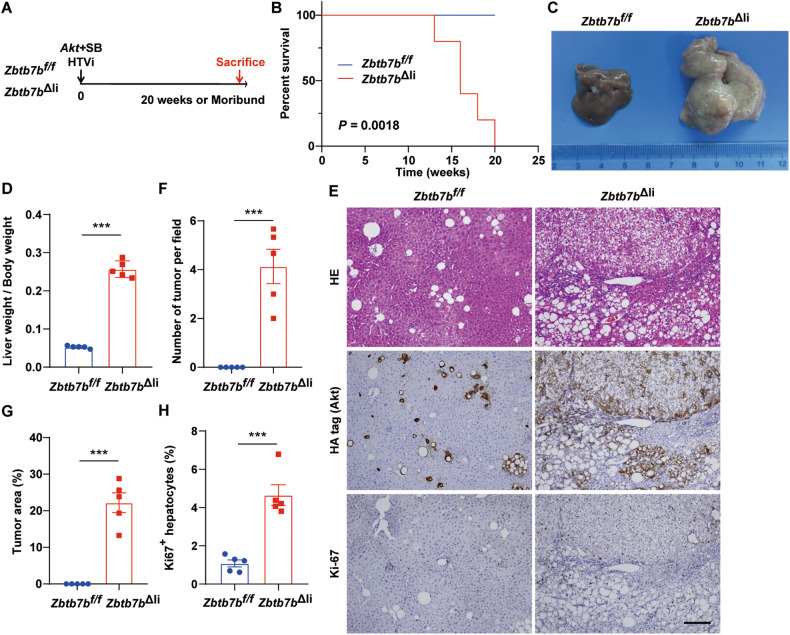
ZBTB7B deficiency primes hepatocytes to Akt-induced liver cancer. **A** Study design. Hydrodynamic tail vein injection (HTVi) of Akt to induce tumor development in *Zbtb7b*^*f/f*^ or *Alb*-*Cre*, *Zbtb7b*^*f/f*^ (*Zbtb7b*^Δli^) mice. Mice were sacrificed 20 weeks (*Zbtb7b*^*f/f*^) after Akt injection or moribund (*Zbtb7b*^Δli^). *n* = 5. **B** Kaplan–Meier survival analysis. **C** Gross liver images of *Zbtb7b*^*f/f*^ and *Zbtb7b*^Δli^ mice 20 weeks after Akt injection. **D** Liver/body weight ratio of *Zbtb7b*^*f/f*^ and *Zbtb7b*^Δli^ mice. **E** H&E staining and immunohistochemistry of HA-tag and Ki67 on liver sections of *Zbtb7b*^*f/f*^ and *Zbtb7b*^Δli^ mice. Magnification: ×10. Scale bar: 200 μm. **F**, **G** Numbers of tumors (**F**) and percentage of tumor area (**G**) in the livers of *Zbtb7b*^*f/f*^ and *Zbtb7b*^Δli^ mice. **H** Percentage of Ki67^+^ hepatocytes in the livers of *Zbtb7b*^*f/f*^ and *Zbtb7b*^Δli^ mice. Data are presented as mean ± SEM. Statistical analyses were performed with the Log-Rank test (**B**) or two-tailed unpaired student’s *t* test (**D**, **F**, **G**, **H**). ***P < 0.001.

Multiple genetic or epigenetic alternations are necessary for hepatocyte transformation. Single oncogene overexpression or tumor suppressor deletion is often insufficient to initiate liver cancer, e.g., activated Akt1 alone is incompetent in inducing liver cancer development (ref. [26]). As ZBTB7B deficiency primed the livers to the fetal-like state and accelerated Akt/N-Ras-induced liver cancer initiation, we next sought to investigate whether ZBTB7B-deficient hepatocytes would be susceptible to single oncogene-induced liver cancer. Consistent with previous reports, no overt tumor formation was observed in the *Zbtb7b*^*f/f*^ mice up to 20 weeks after transducing Akt (Fig. 3A–C). At 20 weeks after Akt oncogene transfer, *Zbtb7b*^*f/f*^ livers were largely normal in size, shape, and color (Fig. 3C–E). No tumor was noticeable on the liver surface (Fig. 3C). Histological inspection revealed a few small preneoplastic lesions in the *Zbtb7b*^*f/f*^ livers (Fig. 3E–G). Immunostaining with HA-tag further confirmed only sporadic Akt-expressing hepatocytes with enlarged clear cytoplasm were present in the *Zbtb7b*^*f/f*^ livers (Fig. 3E). Akt expression in *Zbtb7b*^*f/f*^ livers had minimal effect on hepatocyte proliferation (Fig. 3E, H). In sharp contrast to the *Zbtb7b*^*f/f*^ livers, Akt robustly induced tumor development in the livers of the *Zbtb7b*^Δli^ mice with a median survival of 16 weeks (Fig. 3A–C). *Zbtb7b*^Δli^ livers were pale in color with large tumors on the liver surface (Fig. 3C). Liver size was significantly augmented in *Zbtb7b*^Δli^ mice (Fig. 3C, D). Histological inspection revealed large and multifocal tumors and large numbers of exogenous Akt-expressing and proliferating cells in the *Zbtb7b*^Δli^ livers (Fig. 3E–H).

### c-Jun is a core signaling node in accelerated liver cancer initiation in ZBTB7B-deficient hepatocytes

**Fig. 4:**
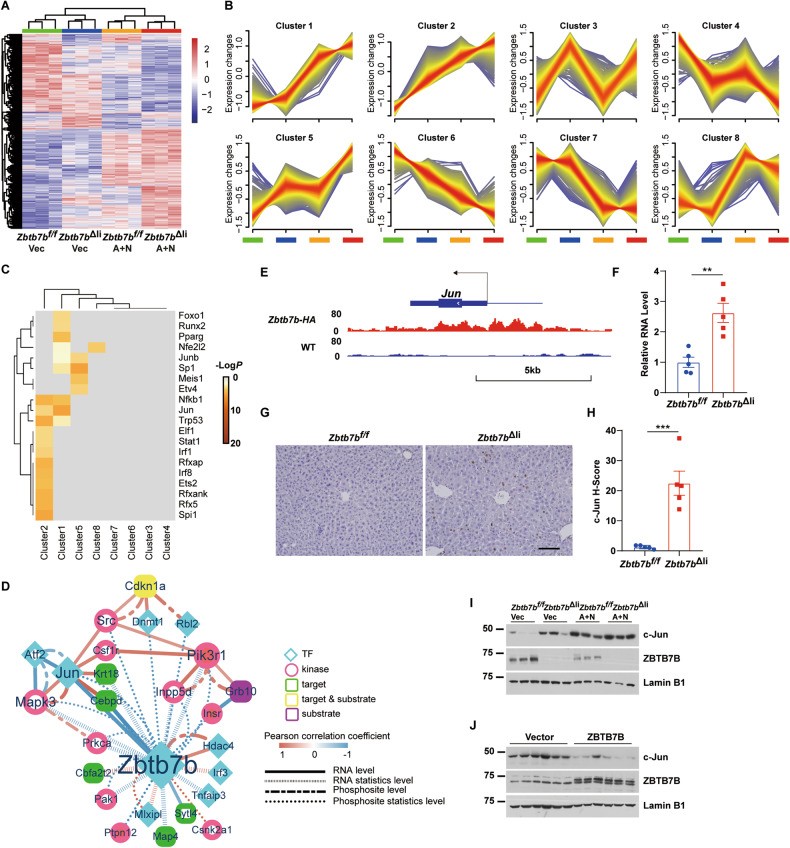
c-Jun is a core node in ZBTB7B-deficient liver cancer initiation. **A** Hierarchical clustering analysis for differentially expressed genes (DEGs). **B** Temporal expression profiling of the DEGs using Mfuzz. **C** TRRUST analysis of transcriptional regulatory relationships. Top 20 transcription factors are shown. **D** Protein-protein interaction network synergistically regulated by ZBTB7B and Akt/N-Ras oncogenes. The shape and color of nodes represent the functional annotation of the gene. Mint blue diamonds: transcription factors, pink ellipses: kinases, dark green round rectangles: targets, purple round rectangles: substrates, yellow round rectangles: targets and substrates. Closed nodes represent direct targets of ZBTB7B. The edges represent the Pearson correlation coefficient calculated based on gene expression. The line type of edges indicated the data type. Solid line: RNA level, dash-dot line: phosphosite level. The color and width of the edges indicate the strength of the correlation. Blue to red: correlation coefficient from −1 to 1. Node size: vitality of the gene. **E** Genome view of the occupancy of ZBTB7B in *Jun* locus. **F** Quantitative RT-PCR analysis of c-Jun expression in *Zbtb7b*^*f/f*^ and *Alb*-*Cre*, *Zbtb7b*^*f/f*^ (*Zbtb7b*^Δli^) livers. *n* = 5. **G** Immunohistochemistry of c-Jun on liver sections of *Zbtb7b*^*f/f*^ and *Zbtb7b*^Δli^ mice. Magnification: ×20. Scale bar: 100 μm. **H** Quantitation of c-Jun immunohistochemistry. *n* = 5. **I** Western blot analysis of c-Jun in liver lysates of *Zbtb7b*^*f/f*^ and *Zbtb7b*^Δli^ mice 2 weeks after injection with vector or Akt/N-Ras. **J** Western blot analysis of c-Jun in liver lysates of mice 4 weeks after Akt/N-Ras injection with GFP or ZBTB7B. Data are presented as mean ± SEM. Statistical analyses were performed with two-tailed unpaired student’s *t* test. **P < 0.01, ***P < 0.001.

8804 genes were differentially expressed between the *Zbtb7b*^*f/f*^ and *Zbtb7b*^Δli^ livers during Akt/N-Ras oncogene-induced hepatocyte transformation (Fig. 4A). Clustering analysis of differentially expressed genes (DEGs) expression tendency identified 8 gene clusters (Fig. 4B and Table S1). Clusters 2, 4, 5, and 6 included genes synergistically regulated by ZBTB7B and Akt/N-Ras oncogenes that were upregulated (clusters 2 and 5) or downregulated (clusters 4 and 6) in the *Zbtb7b*^Δli^ livers and further upregulated or downregulated upon Akt/N-Ras oncogene expression (Fig. 4B and Table S1). Whereas genes in clusters 1, 7, and 8 were mainly regulated by Akt/N-Ras oncogenes (Fig. 4B and Table S1). Transcription factor analysis showed that genes in each cluster were regulated by different sets of transcription factors, especially those involved in liver cancer development, including c-Jun, NF-κB, STAT1, and P53 (Fig. 4C and Table S2). KEGG pathway enrichment analysis suggested regulation of cell cycle, Ras-MAPK signaling, PI3K-Akt signaling, chemokine signaling, and NF-κB pathway were differentially activated (Fig. S5A). Quantitative gene set variation analysis (GSVA) further revealed Ras-MAPK, PI3K-Akt, and Hippo signaling were partially activated in the *Zbtb7b*^Δli^ livers that were further activated upon Akt/N-Ras oncogene expression (Fig. S5B). Global phosphoproteomic profiling revealed 8459 differentially expressed phosphosites (DEPs) classed into 8 clusters (Fig. S5C, D, and Table S3). Cluster 2 DEPs represented phosphosites enriched in the *Zbtb7b*^Δli^ livers, including phosphosites on P21, c-Jun, and Ki67, which were significantly upregulated upon Akt/N-Ras oncogene expression (Fig. S5D and Table S3). NetworKIN analysis (ref. [27]) enriched activities of 68 kinases from DEPs (Fig. S5E and Table S4). Transcription factor analysis enriched 13 TFs at the phosphoproteomic level (Table S2). To investigate the key regulators responsible for accelerated liver cancer initiation in the ZBTB7B-deficient livers, protein-protein interaction (PPI) network was constructed by integrated analysis of the functional intersection of DEGs and DEPs. 122 nodes and 368 edges were identified (Fig. S5F and Table S5). Functional genes, e.g., *Akt1*, *Shc1*, *Prkce*, *Jun*, and *Hdac1*, were highly related to each other and might play important regulatory roles in the accelerated liver cancer initiation in *Zbtb7b*^Δli^ livers (Fig. S5F and Table S5).

To investigate which nodes were directly regulated by ZBTB7B, chromatin immunoprecipitation (ChIP)-sequencing was performed on the livers of the *Zbtb7b*-*HA* mice with an HA-tag in frame knocked-in before the stop codon of *Zbtb7b* gene (Fig. S6A, B). ZBTB7B is bound mainly to the promoter region vicinity of the transcription start site (Fig. S6C and S6D). ZBTB7B bound to the promoters of known ZBTB7B targets genes, e.g., *Irs1* (ref. [15]), as well as genes important in liver cancer, including *Jun*, *Akt1*, *Ccnd1*, and *Mki67* (Table S6). Functional enrichment analysis of ZBTB7B target genes suggested that ZBTB7B played a more prominent role in pathways in metabolism and cancer (Fig. S6E). Integration of ZBTB7B direct target genes into the PPI network revealed *Jun*, *Akt1*, *Shc1*, and *Prkce* as the top nodes with the highest degree correlation to ZBTB7B (Fig. S6F). PPI sub-network of DEGs and DEPs synergistically regulated by ZBTB7B and Akt/N-Ras oncogens (clusters 2, 4, 5, and 6) further revealed c-Jun as the central signaling node in accelerated liver cancer initiation in the *Zbtb7b*^Δli^ livers (Fig. 4D). AP1 family transcription factor c-Jun, first identified as viral oncoprotein (ref. [28]), is required in diethylnitrosamine (DEN)-induced liver cancer initiation (ref. [29, 30]). c-Jun, identified in the cluster 2 DEGs (Fig. 4B and Table S1), was a direct target gene of ZBTB7B that ZBTB7B is directly bound to the *Jun* promoter (Fig. 4E). c-Jun expression was significantly increased in the *Zbtb7b*^Δli^ livers (Fig. 4F–H). Expression of Akt/N-Ras oncogenes downregulated ZBTB7B expression and increased c-Jun expression (Fig. 4I). Increased c-Jun expression upon Akt/N-Ras expression was attenuated by ectopic ZBTB7B expression (Fig. 4J).

### c-Jun is essential to accelerated hepatocarcinogenesis in ZBTB7B-deficient hepatocytes

**Fig. 5:**
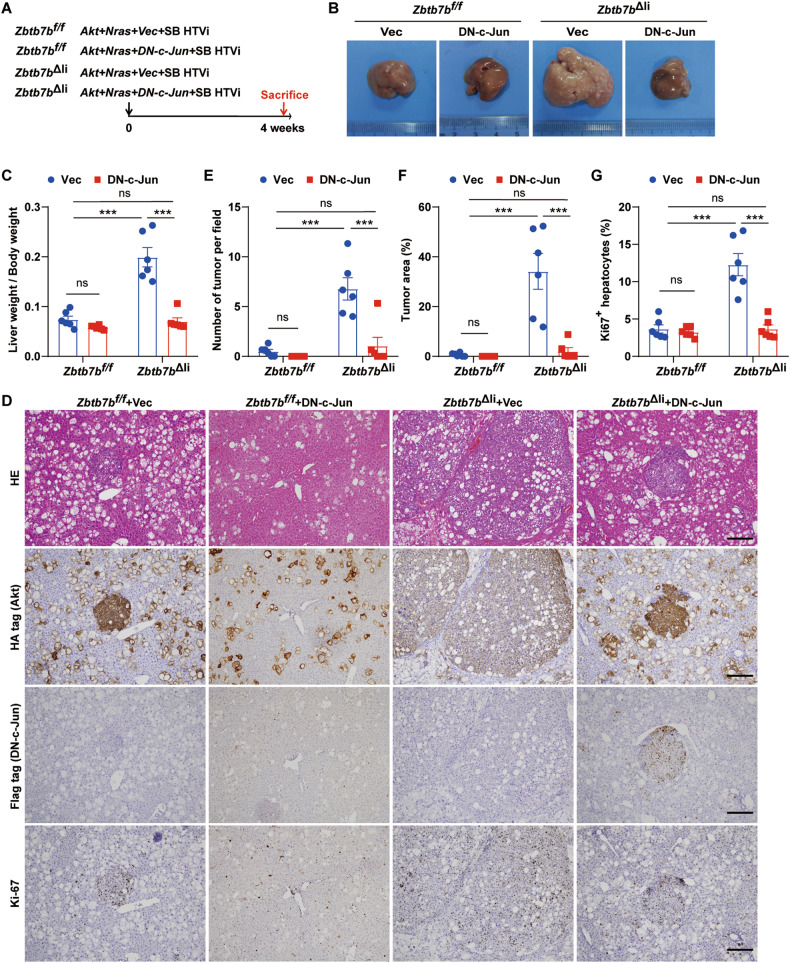
c-Jun is essential to accelerated liver cancer development in ZBTB7B-deficient livers. **A** Study design. Hydrodynamic tail vein injection (HTVi) of Akt/N-Ras to induce tumor development in *Zbtb7b*^*f/f*^ or *Alb*-*Cre*, *Zbtb7b*^*f/f*^ (*Zbtb7b*^Δli^) mice with pT3-N-FLAG vector control (Vec) or DN-c-Jun. Mice were sacrificed 4 weeks after oncogene injection. *n* = 6. **B** Gross liver images of *Zbtb7b*^*f/f*^ and *Zbtb7b*^Δli^ mice. **C** Liver/body weight ratio of *Zbtb7b*^*f/f*^ and *Zbtb7b*^Δli^ mice. **D** H&E staining and immunohistochemistry of HA-tag, FLAG-tag, and Ki67 on liver sections. Magnification: ×10. Scale bars: 200 μm. **E**, **F** Numbers of tumors (**E**) and percentage of tumor area (**F**). **G** Percentage of Ki67^+^ hepatocytes. Data are presented as mean ± SEM. Statistical analyses were performed with two-way ANOVA followed by Šídák’s multiple comparison test. ***P < 0.001. ns: not significant.

As c-Jun was identified as a core signaling node in Akt/N-Ras-induced ZBTB7B-deficient liver cancer initiation (Fig. 4), we next sought to investigate whether ZBTB7B deficiency accelerates liver cancer initiation through c-Jun. Truncated c-Jun, which lacked the N-terminal transactivation domain, exerted dominant negative effects on c-Jun-mediated transcription (ref. [31]). Ectopic dominant negative (DN)-c-Jun expression inhibited Akt/N-Ras-induced tumor development in the *Zbtb7b*^Δli^ livers (Fig. 5A, B) with significantly reduced liver/body weight ratio to the extent comparable to the *Zbtb7b*^*f/f*^ livers (Fig. 5C). Histological inspection of DN-c-Jun-expressing *Zbtb7b*^Δli^ livers revealed much-reduced tumor numbers, tumor area and Ki67^+^ proliferative cell numbers to the levels of the *Zbtb7b*^*f/f*^ livers (Fig. 5D–G). The essential roles of c-Jun in accelerated hepatocarcinogenesis were further demonstrated by knocking down c-Jun expression in ZBTB7B-deficient hepatocytes (Fig. S7). ZBTB7B deficiency was permissive to single oncogene Akt-induced hepatocarcinogenesis (Fig. 3). DN-c-Jun expression completely abolished Akt-induced liver cancer development in the *Zbtb7b*^Δli^ livers (Fig. S8), suggesting c-Jun is downstream of ZBTB7B mediating hepatocyte transformation and liver cancer development.

### ZBTB7B negatively regulates c-Jun function in hepatocarcinogenesis

**Fig. 6:**
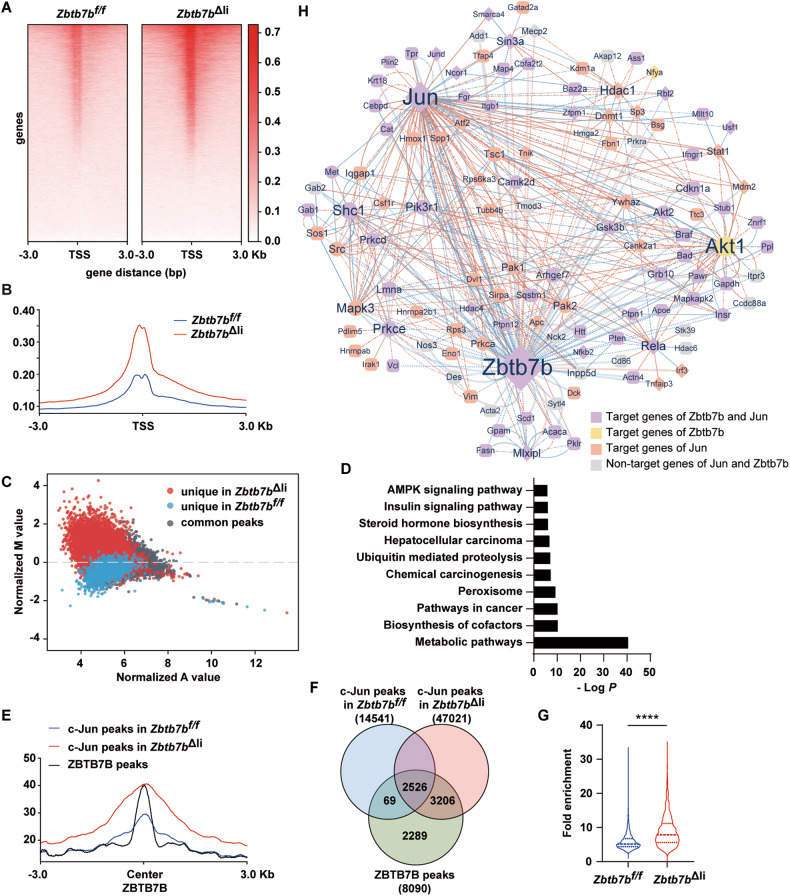
ZBTB7B negatively regulates c-Jun function in hepatocarcinogenesis. **A**–**G** ChIP-seq analysis of genome-wide c-Jun occupancy in *Zbtb7b*^*f/f*^ and *Alb*-*Cre*, *Zbtb7b*^*f/f*^ (*Zbtb7b*^Δli^) livers. **A**, **B** Heatmaps (**A**) and distribution of c-Jun binding intensity (**B**) centered at transcription start site (TSS). **C** Mean-average (MA) plot of differential c-Jun ChIP-seq peak density in the *Zbtb7b*^*f/f*^ and *Zbtb7b*^Δli^ livers. Log fold change (M) and log of the mean (A) of normalized read density are shown. **D** KEGG pathway analysis of genes corresponding to ChIP-seq peaks unique in the *Zbtb7b*^Δli^ livers. **E** Enrichment profiles representing the c-Jun ChIP-seq reads density from the *Zbtb7b*^*f/f*^ and *Zbtb7b*^Δli^ livers centered at ZBTB7B peaks. **F** Venn diagram showing the overlaps among c-Jun peaks from *Zbtb7b*^*f/f*^ and *Zbtb7b*^Δli^ livers and ZBTB7B peaks. **G** c-Jun ChIP signals from the 2526 c-Jun/ZBTB7B sites in the *Zbtb7b*^*f/f*^ and *Zbtb7b*^Δli^ livers. **H** Protein-protein interaction network synergistically regulated by ZBTB7B and c-Jun. Node color denotes the upstream transcription factors. The shape of the nodes, the color, and the line type of the edges are the same as in Fig. 4D. Data are presented as mean ± SEM. Statistical analyses were performed with two-tailed paired student’s *t* test. ****P < 0.0001.

To determine the mechanistic role of c-Jun in mediating accelerated liver cancer initiation in *Zbtb7b*-deficient hepatocytes, ChIP-seq analysis of c-Jun genome-wide binding was performed. *Zbtb7b* deficiency led to significantly increased global c-Jun occupancy (Fig. 6A, B, Table S7 and S8). Upon ZBTB7B deletion, additional c-Jun binding peaks were detected in genes involved in regulating metabolism and cancer (Fig. 6C, D). We then investigated whether ZBTB7B can directly interfere with c-Jun chromatin occupancy by analyzing the relative position of the c-Jun and ZBTB7B-binding sites and revealed that the c-Jun peaks in both *Zbtb7b*^*f/f*^ and *Zbtb7b*^Δli^ livers largely overlapped with the corresponding ZBTB7B peaks in the wild-type livers (Fig. 6E). Interestingly, 32.1% (2595 of 8090) of ZBTB7B-binding sites overlapped with the c-Jun binding sites in the *Zbtb7b*^*f/f*^ livers, while the vast majority of ZBTB7B-binding sites (70.9%, 5732 of 8090) overlapped with the c-Jun binding sites in the *Zbtb7b*^Δli^ livers (Fig. 6F). In addition, c-Jun occupancy was significantly increased on 2526 c-Jun/ZBTB7B loci in the *Zbtb7b*^Δli^ livers, compared to the *Zbtb7b*^*f/f*^ livers (Fig. 6G), suggesting ZBTB7B directly antagonizes c-Jun chromatin occupancy. Furthermore, 50% of the nodes in the PPI network, including *Zbtb7b*, *Jun*, and *Shc1*, were co-regulated by ZBTB7B and c-Jun (Fig. 6H and Table S5).

**Fig. 7:**
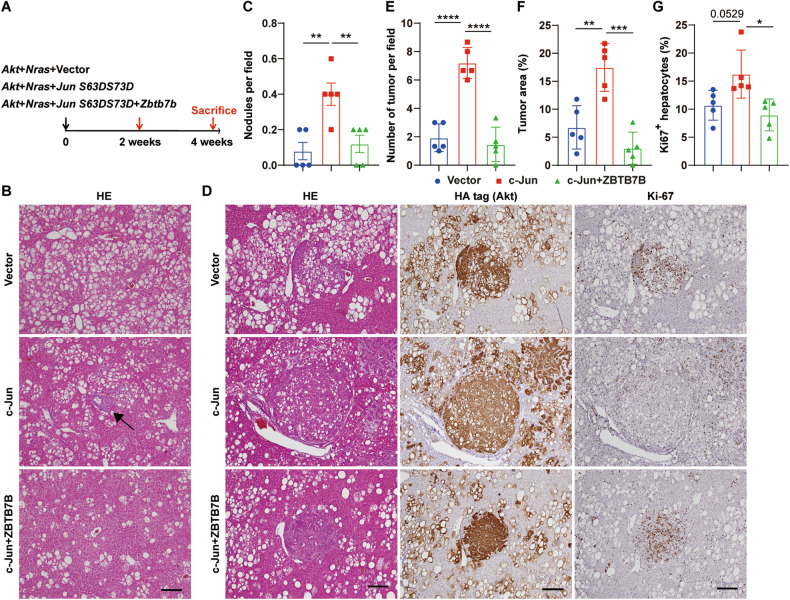
ZBTB7B attenuates the tumor-promoting functions of c-Jun. **A** Study design. Hydrodynamic tail vein injection (HTVi) of Akt and N-Ras oncogenes with active c-Jun (c-Jun S63D/S73D) and ZBTB7B. Mice were sacrificed 2 weeks or 4 weeks after hydrodynamic injection. *n* = 5. **B** H&E staining on liver sections of wild-type mice 2 weeks after Akt/N-Ras injection with active c-Jun and ZBTB7B. Magnification: ×10. Scale bar: 200 μm. **C** Numbers of tumor nodules 2 weeks after Akt/N-Ras injection with active c-Jun and ZBTB7B. **D** H&E staining and immunohistochemistry of HA-tag and Ki67 on liver sections of wild-type mice 4 weeks after Akt/N-Ras injection with active c-Jun and ZBTB7B. Magnification: ×10. Scale bars: 200 μm. **E**–**G** Numbers of tumors (**E**), percentage of tumor area (**F**), and Ki67^+^ hepatocytes (**G**) in the livers 4 weeks after Akt/N-Ras injection with active c-Jun and ZBTB7B. Data are presented as mean ± SEM. Statistical analyses were performed with one-way ANOVA followed by Tukey’s multiple comparison test. *P < 0.05, **P < 0.01, ***P < 0.001, ****P < 0.0001.

We next sought to investigate whether ZBTB7B suppresses liver cancer development by blocking c-Jun function, in addition to regulating c-Jun expression. Phosphorylation of the c-Jun transactivation domain on serines 63 and 73 increases its transcription activity (ref. [32, 33]). Expression of active c-Jun (c-Jun S63D/S73D) accelerated Akt/N-Ras oncogene-induced liver cancer initiation (Fig. 7A–C) and liver cancer development (Fig. 7D–G). In contrast to more preneoplastic nodules at 2 weeks (Fig. 7B, C) and more tumors and proliferative hepatocytes at 4 weeks after active c-Jun expression (Fig. 7D–G), simultaneous overexpression of ZBTB7B completely abolished the tumor-promoting effects of active c-Jun (Fig. 7). ZBTB7B overexpression reduced the numbers of preneoplastic nodules, tumor numbers, tumor area, and Ki67^+^ proliferative hepatocyte numbers to the levels of vector control (Fig. 7).

### ZBTB7B signature is associated with liver cancer prognosis

**Fig. 8:**
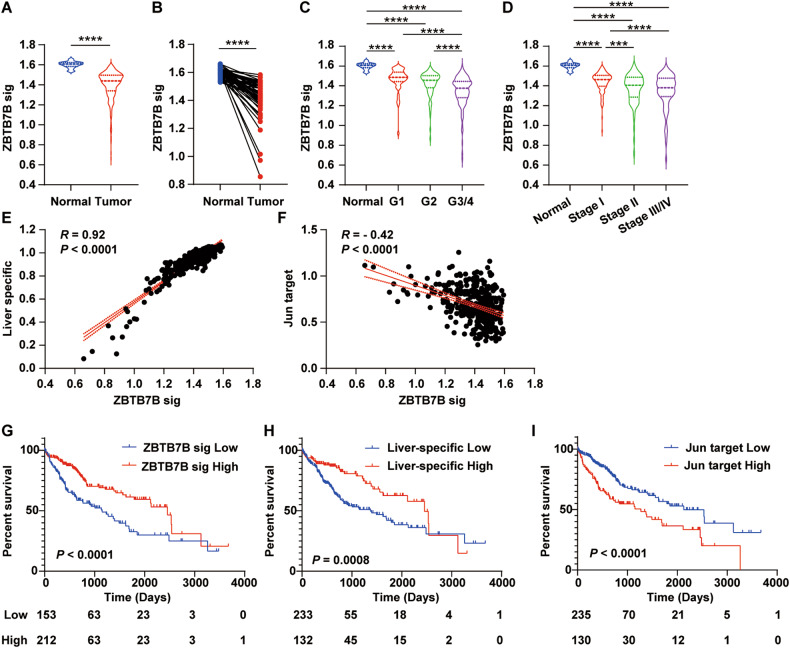
ZBTB7B signature predicts liver cancer prognosis. Gene set variation analysis (GSVA) scores of ZBTB7B signature genes, liver-specific genes (HSIAO_LIVER_SPECIFIC_GENES), and c-Jun target genes (MATTHEWS_AP1_TARGETS) were calculated for each HCC patient samples in the TCGA_LIHC cohort. **A** Comparison of ZBTB7B signature scores in tumors and adjacent normal tissues. **B** Comparison of ZBTB7B signature scores in paired tumors and adjacent normal tissues. **C**, **D** Comparison of ZBTB7B signature scores in HCC tumors of different grades (**C**) and stages (**D**). **E**, **F** Pearson correlation of ZBTB7B signature scores to expression levels of liver-specific genes (**E**) and c-Jun target genes (**F**). **G**–**I** Probabilities of overall survival of HCC patients according to the expression level of the ZBTB7B signature (**G**), liver-specific genes (H), and c-Jun target genes (**I**). Statistical analyses were performed with two-tailed unpaired (**A**) or paired (**B**) student’s *t* test, one-way ANOVA followed by Tukey’s multiple comparison tests (**C**, **D**), or Log-Rank test (**G**–**I**). ***P < 0.001, ****P < 0.0001.

To determine to which extent ZBTB7B might be associated with human liver cancer phenotypes, we queried the Cancer Genome Atlas hepatocellular carcinoma (TCGA_LIHC) dataset. 1105 of 2166 genes with overlapping ZBTB7B and c-Jun binding were differentially expressed in which 176 genes were downregulated in the *Zbtb7b*^Δli^ livers and further downregulated upon Akt/N-Ras oncogene expression (cluster 4 and 6, *P* < 0.001) (Fig. S9A). 140 human orthologous genes were used as ZBTB7B signature to assess the ZBTB7B activity in liver cancer samples (Fig. S9A and Table S9). Expression of the 140-gene ZBTB7B signature was significantly lower in HCC tumors (Fig. 8A). Comparison of paired tumor samples and adjacent normal liver tissues further supported that ZBTB7B activity was downregulated in the liver cancer (Fig. 8B). ZBTB7B activity was significantly lower in all stage/grade liver cancers, compared to the adjacent normal tissues (Fig. 8C, D). A comparison of liver cancers at different stages and grades revealed that advanced liver cancer only had a moderate further decrease of ZBTB7B activity (Fig. 8C, D). Association and linear regression analyses demonstrated a tight correlation between ZBTB7B signature and liver-specific gene expression levels in human HCC (Fig. 8E), consistent with the finding that ZBTB7B was an adult liver-enriched transcription factor (Fig. S1) and expression of adult liver-specific genes were significantly downregulated in the *Zbtb7b*^Δli^ livers (Fig. S1B). In line with increased expression and activity of c-Jun in the *Zbtb7b*^Δli^ livers (Fig. 4), ZBTB7B signature and c-Jun target gene expression levels negatively correlated to each other in human HCCs (Fig. 8F). Patients with high ZBTB7B signature (HR: 0.50 (0.35–0.71), *P* < 0.0001) and high liver-specific gene expression (HR: 0.52 (0.36–0.73), *P* = 0.0008) had better prognosis, while patients with high c-Jun target gene expression (HR: 1.94 (1.34–2.81), *P* < 0.0001) had significantly shorter overall survival (Fig. 8G–I). In a second cohort of 244 liver cancer patients, ZBTB7B activity was similarly lower in the liver cancers compared to the adjacent normal tissues (Fig. S9B, C), but comparable among different stages (Fig. S9D–F). ZBTB7B signature was correlated to the liver-specific gene expression levels but negatively correlated to the c-Jun target gene expression (Fig. S9G, H). ZBTB7B signature and liver-specific genes predicted a good prognosis, while c-Jun target genes predicted a poor prognosis (Fig. S9I–K).

## Discussion

In this study, we identify transcription factor ZBTB7B as a suppressive regulator of HCC initiation. ZBTB7B-deficient hepatocytes are susceptible to oncogenic transformation and HCC initiation. ZBTB7B suppresses HCC initiation by inhibiting c-Jun expression and activity, a key transcription factor in regulating HCC initiation.

ZBTB7B is a potential permissive regulator in HCC initiation. Hepatocarcinogenesis is the consequence of the accumulation of genetic and epigenetic alterations (ref. [1, 5]). Co-occurrence of functional driver mutations provides liver cancer cells growth advantage (ref. [5]). These gene mutations alter key signaling pathways in liver cancer cells, e.g., telomere maintenance, Wnt/β-catenin, P53/cell cycle regulation, Akt/mTOR, MAP kinase and oxidative stress (ref. [7, 34, 35]). However, activation of a single signaling pathway is generally insufficient in inducing HCC development. Overexpression of activated Akt1 can only induce liver cancer development with very long latency in the mouse models (ref. [26]). In contrast, Akt1 robustly induces HCC development in the background of ZBTB7B deficiency with much-shortened latency. Although mutation or deletion of *ZBTB7B* is not a frequent event in HCC, ZBTB7B expression is downregulated at the early-stage of Akt/N-Ras-induced HCC. Epigenetic dysregulation plays a crucial role in hepatocarcinogenesis (ref. [1, 5]). Expression and/or transcription activity of ZBTB7B might be regulated at early stages in hepatocarcinogenesis which provides hepatocytes permissive signals to oncogenic transformation. Consistent with this notion, genes regulated by ZBTB7B are already downregulated in very early-stage HCC (Fig. 8 and S9).

Transcriptomic profiling of HCC tumors reveals a subset of HCCs that are less differentiated expressing progenitor markers IGF2, EpCAM, and AFP (ref. [36–38]). Hepatocytes might dedifferentiate into progenitor-like cells before transforming into HCC (ref. [39, 40]). ZBTB7B is an adult liver-enriched transcription factor and is associated with adult liver features. In line with the functions as a cell lineage commitment regulator in multiple developmental processes (ref. [12–14]), ablation of ZBTB7B attenuates adult liver-specific gene expression (Fig. 1). Interestingly, the ZBTB7B gene signature (Fig. S9 and Table S9) significantly overlaps with the signatures of hepatoblastoma (ref. [41]) (42 of 140 in ZBTB7B gene signature, FDR *q* value 4.69E-26), liver-specific genes (ref. [16]) (23 of 140 in ZBTB7B gene signature, FDR q-value 1.32E-22), HCC subclass characterized by increased proliferation, high AFP levels and chromosomal instability (ref. [42]) (18 of 140 in ZBTB7B gene signature, FDR *q* value 5.61E-18), and HCC subtype associated with hepatocyte differentiation (ref. [37]) (18 of 140 in ZBTB7B gene signature, FDR *q* value 3.30E-15). Attenuated ZBTB7B expression and/or transcriptional activity might therefore maintain hepatocytes at the dedifferentiated state that is more susceptible to oncogenic transformation and HCC development.

Integrated analyses reveal that c-Jun, a direct target gene of ZBTB7B, functions as the core signaling node in ZBTB7B-deficient HCC initiation (Fig. 4D). c-Jun is required in DEN-induced mouse liver cancer initiation (ref. [29, 30]). c-Jun activation is predicted to play key roles in the development of a hepatoblast subtype HCC arising from hepatic progenitor cells (ref. [43]). ZBTB7B not only regulates c-Jun expression, but also regulates c-Jun transcriptional activity in the early-stage of Akt/N-Ras-induced HCC. The majority of ZBTB7B-binding sites in the genome overlap with c-Jun binding sites and ZBTB7B deficiency increases c-Jun chromatin binding (Fig. 6). It is noteworthy that 50% of the signaling nodes in the regulatory network are co-regulated by ZBTB7B and c-Jun (e.g., *Insr*, *Pik3r1*, *Shc*, and *Braf*), and about one-third of the signaling nodes are genes directly regulated by c-Jun, but not ZBTB7B (e.g., *Mapk3*, *Iqgap1*, *Sos*, *Tsc1*, and *Rps6ka3*) (Fig. 6H). The signaling nodes also contain genes regulated by transcription factors other than ZBTB7B and c-Jun, e.g., *Mecp2*, which binds to methylated DNA and mediates transcriptional repression through interaction with HDACs and the SIN3A corepressor. Ras/MAPK and PI-3 kinase/Akt signaling were substantially more activated in the *Zbtb7b*-deficient livers upon Akt/N-Ras oncogene expression (Fig. S5B), suggesting that ZBTB7B may regulate HCC initiation at least partly by regulating MAPK and Akt function in a c-Jun-dependent manner. ZBTB7B deficiency also significantly increases the activity of NF-κB and STAT1, key transcription factors mediating inflammatory responses (Fig. S6F). Chronic liver inflammation and immune regulation play important roles in HCC development (ref. [44]). Inflammatory cytokines, e.g., IL-6 and TNF-α, synergistically regulate hepatocyte transformation and HCC development (ref. [45, 46]). Damage-associated molecular patterns released from damaged hepatocytes activate innate immunity signaling in hepatocytes and the microenvironment and accelerate HCC development (ref. [44]). ZBTB7B may therefore orchestrate c-Jun-inflammatory signal crosstalk and regulate HCC initiation through a broader network of signaling pathways and effector molecules.

ZBTB7B functions as both a transcriptional activator and repressor by interaction with different epigenetic regulators. ZBTB7B interacts with class I (HDACs 1, 2, 3) and class II (HDACs 4, 5, 10) HDACs (ref. [47, 48]). ZBTB7B may form multicomponent complexes with different HDAC-interacting transcriptional corepressors, e.g., HDAC1/2-interacting Sin3a and HDAC3-interacting NcoR2 (Fig. S6F) (ref. [48]). HDAC-dependent histone deacetylation is required for ZBTB7B-mediated *Cd8* transcription silencing during CD4 T cell differentiation (ref. [47]). ZBTB7B also interacts with acetyltransferase p300 and KAT5 (ref. [49, 50]) and may promote histone acetylation and gene transcription at a subset gene loci. p300 catalyzes histone crotonylation and succinylation (ref. [51, 52]), while Class I HDACs are major histone decrotonylases and desuccinylases (ref. [53, 54]). It raises the interesting possibility that ZBTB7B may regulate gene transcription by modulating histone crotonylation and succinylation, which play significant roles in regulating chromatin structure and gene transcription. Besides histone acylation, ZBTB7B may directly or indirectly interact with other epigenetic regulators, e.g., CBX3, CTBP1/2, UHRF1, as well as WDR5 (ref. [48]), which suppress or activate gene transcription. ZBTB7B is a multi-domain protein. The N-terminal BTB/POZ domain is responsible for ZBTB7B dimerization and interaction with transcriptional co-regulators, while the C-terminal zinc finger domains are responsible for DNA binding. Both BTB/POZ and zinc finger domains are required for ZBTB7B-mediated transcriptional regulation and function (ref. [47, 55]). It warrants further investigation whether ZBTB7B interacts with different epigenetic regulators and transcriptional co-regulators, which may result in changes of histone modification and chromatin accessibility to other transcription factors, e.g., c-Jun, and ultimately result in gene transcriptional activation or repression in a gene loci-specific manner.

In summary, ZBTB7B is an adult hepatocyte-enriched transcription factor and is a permissive regulator of HCC initiation. ZBTB7B deficiency primes hepatocytes to a fetal state, sensitizes hepatocytes to oncogenic transformation, and accelerates HCC initiation. ZBTB7B exerts tumor-suppressive functions by directly regulating c-Jun expression and competing with c-Jun for chromatin binding.

## Materials and methods

### Animal experiments

All mice were maintained in a C57/BL6 background and housed in a specific pathogen-free environment and treated in strict accordance with protocols approved by the Institutional Animal Care and Use Committee (Approval number: SIBCB-NAF-15-003-S325-006). *Zbtb7b*^*f/f*^ mice (ref. [56]) were crossed with *Alb*-*cre* mice to generate *Alb*-*Cre*, *Zbtb7b*^*f/f*^ (*Zbtb7b*^Δli^) mice. *Zbtb7b*-*HA* knockin mice were generated as described (ref. [57]) by the Genome Tagging Project (GTP) Center at the Center for Excellence in Molecular Cell Science, Chinese Academy of Sciences. pT3-myr-Akt1-HA, pT3-N-Ras V12, and sleeping beauty transposase (SB) expression plasmids were generously provided by Dr. Xin Chen (UCSF). The coding sequences of *Zbtb7b* and *Jun* were amplified using mouse liver cDNA as a template and cloned into a pT3 vector. 6–8-week-old male wild-type, *Zbtb7b*^*f/f*^, and *Zbtb7b*^Δli^ mice were randomly allocated into groups and hydrodynamic injection was performed to induce liver cancer as described (ref. [17, 58]). No blinding was performed.

### Histology and immunohistochemistry

Mice were anesthetized and livers were isolated and fixed in 4% paraformaldehyde followed by embedding in paraffin. Paraffin-embedded livers were sectioned and stained with hematoxylin and eosin (H&E) or subjected to immunohistochemical staining as previously described (ref. [59]). The primary antibodies used are listed in Supplementary Table S10. Images were captured at 3 random fields at ×4, ×10, ×20, or ×40 magnifications with a digital camera (DP71; OLYMPUS, Inc.) and Digital Acquire software (DPController; OLYMPUS, Inc.).

### RNA-seq and ChIP-seq analyses

Total RNA was extracted and purified from livers using TRIZOL (Invitrogen). Three biological replicates were subjected to complementary DNA library preparation and sequencing according to the Illumina standard protocol. RNA-seq data was mapped to the mouse mm10 reference genome by STAR (v.2.9). R package DESeq2 (v.1.24.0) was used to perform normalization and differential expression analysis. Gene ontology analysis was performed using g:profiler (ref. [60]). GSEA was performed on the GSEA application (v.4.0.3) with gene signatures obtained from the MSigDB database (v7.5.1). Statistical significance was assessed by comparing the enrichment score to enrichment results generated from 1,000 random permutations of the gene set to obtain a *P*-value (nominal *P*-value) (ref. [61]).

Perfused and formaldehyde cross-linked liver tissues were homogenized and cells were collected by centrifugation. Chromatin at an average size of 200–500 bp was incubated with HA antibody, c-Jun antibody, or normal rabbit IgG. Immunoprecipitated chromatin DNA was subjected to library preparation using a library preparation kit (#ND607, Vazyme) according to the manufacturer’s instructions. The libraries were sequenced on Illumina Novaseq. ChIP-seq data was mapped to the mouse mm10 reference genome by Bowtie2 (v.2.3.1). Peak detection was performed using the MACS2 (v.2.1.1). The downstream visualization and annotation were performed by IGV (v.2.11.2) and R package ChIPseeker (v.1.30.3) respectively. Peak overlapping was computed by using the default bedtools intersect intervals functions with at least 1 base pair overlap. MA plot was generated by MAnorm (v.1.3.0) (ref. [62]).

### Mass spectrometry analysis

Phosphopeptide enrichment in mouse liver was performed according to the previous report (ref. [7]). With a two-hour liquid chromatography gradient elution method, the phosphopeptides were identified in data independent analysis (DIA) mode by Thermo Scientific Q Exactive HF-X Hybrid Quadrupole-Orbitrap mass spectrometer and analyzed using Spectronaut^TM^ 15 (Biognosys Inc.). Peptide Collapse in Perseus (1.6.8.0) (ref. [63]) was used to combine Spectronaut precursor quantifications into consensus phosphosites. The differentially expressed phosphosites (DEPs) were obtained by analysis of variance (ANOVA *P* value < 0.05) and were divided into 8 clusters according to their expression patterns. The kinases estimated by NetworKIN 3.0 (ref. [27]) and transcription factors estimated by TRRUST (Built into Metascape, http://www.metascape.org/) of each cluster were obtained by kinase-substrate enrichment analysis and transcription factor enrichment analysis, respectively. All possible protein-protein interactions (PPI) were obtained using known databases (MIPPIE, TRRUST, UniProt, and STRING), and their Pearson correlation coefficients were calculated. A simplified phosphoproteome-level PPI network was obtained. Transcriptome-level PPI network was treated in the same way. Network relationships were presented by Cytoscape (Version 3.8.0, http://www.cytoscape.org/).

### Patient survival analysis

Mouse liver (PRJNA66167) and human liver (PRJNA280600) transcriptome data were downloaded from GEO and mapped to mm10 and hg19 reference genomes, respectively. Raw counts were normalized to FPKM. R package Mfuzz was used to perform cluster analysis on default parameters. Gene set enrichment scores of ZBTB7B signature (Table S9), Hsiao liver-specific genes (ref. [16]) and Matthews AP1 targets (ref. [64]) were calculated for each sample in previously published hepatocellular carcinoma (TCGA (ref. [35]) and GSE14520 (ref. [65]) gene expression data sets using GSVA (ref. [66]). Survival analysis was performed using the Kaplan–Meier (Log-Rank test) method to evaluate the correlation of the signature GSVA scores with overall survival in these two data sets. ROC analyses were applied to detect the optimal cutoff point.

### Statistical analysis

Variance similarity between the groups is being statistically compared. Data meet the assumptions of the tests. No data were excluded from the analysis. The sample size was based on previous research experience. Sample numbers were indicated in the figure legends. Statistical significance between conditions was assessed by unpaired or paired two-tailed Student’s *t*-tests, one-way ANOVA followed by Tukey’s post-hoc analysis, or two-way ANOVA followed by Šídák’s multiple comparison test using GraphPad Prism. Error bars represent SEM.

### Supplementary information

Supplementary figures and methods

Supplementary tables

Original Data File

Checklist

## Data Availability

RNA-seq and ChIP-seq data are deposited to the National Omics Data Encyclopedia (NODE, https://www.biosino.org/node, accession number OEP003425).
