# Supplementary material for: ZBTB7B is a permissive regulator of hepatocellular carcinoma initiation by repressing c-Jun expression and function
Source: Cell Death Dis. 2024 Jan 15;15(1):55. doi: 10.1038/s41419-024-06441-y (PMC10789742; doi:10.1038/s41419-024-06441-y)
Supplement: Supplementary file 1 — Supplementary figures and methods [file 41419_2024_6441_MOESM1_ESM.docx]

**ZBTB7B is a permissive regulator of hepatocellular carcinoma initiation by repressing c-Jun expression and function**

Yue Zhu^1,#^, Qinqin Wang^2,#^, Xinyu Xie^1^, Cuihong Ma^1^, Yuemei Qiao^1^, Yu Zhang^3^, Yanjun Wu^1^, Yuan Gao^1^, Jing Jiang^4^, Xin Liu^3^, Jianfeng Chen^1,5^, Chen Li^2,^*, Gaoxiang Ge^1,^*

**The supplementary information contains 9 supplementary figures, 11 supplementary tables and supplementary materials and methods.**

**
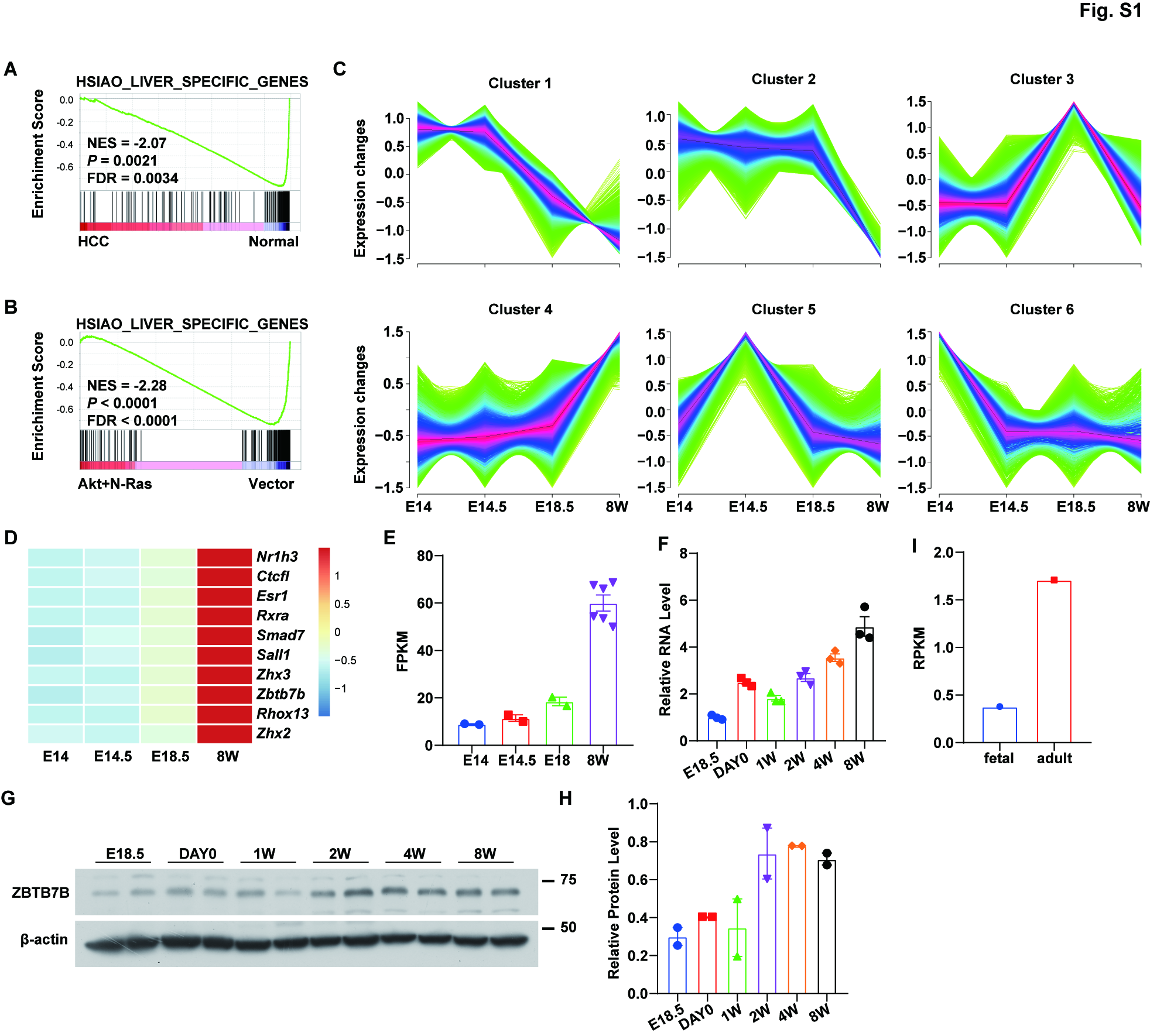
**

**Fig. S1 ZBTB7B is highly expressed in adult hepatocytes.**

(A) GSEA comparison of liver specific gene expression between liver tumors and adjacent normal livers in TCGA cohort.

(B) GSEA comparison of liver specific gene expression between Akt/N-Ras-induced liver tumors and normal adult livers.

(C) Clustered profiles of gene expression in the livers of embryonic day 14 (E14), 14.5 (E14.5), 18.5 (E18.5) and 8-week adult mice.

(D) Expression heat map of top 10 transcription factors in cluster 4.

(E) FPKM of *Zbtb7b* transcription factor in mouse liver development.

(F) Quantitative RT-PCR analysis of *Zbtb7b* transcription factor in mouse liver development. n = 3

(G) Western blot analysis of ZBTB7B transcription factor in mouse liver development. (H) Quantitation of (F).

(I) FPKM of *ZBTB7B* transcription factor in human fetal liver and adult liver.

**
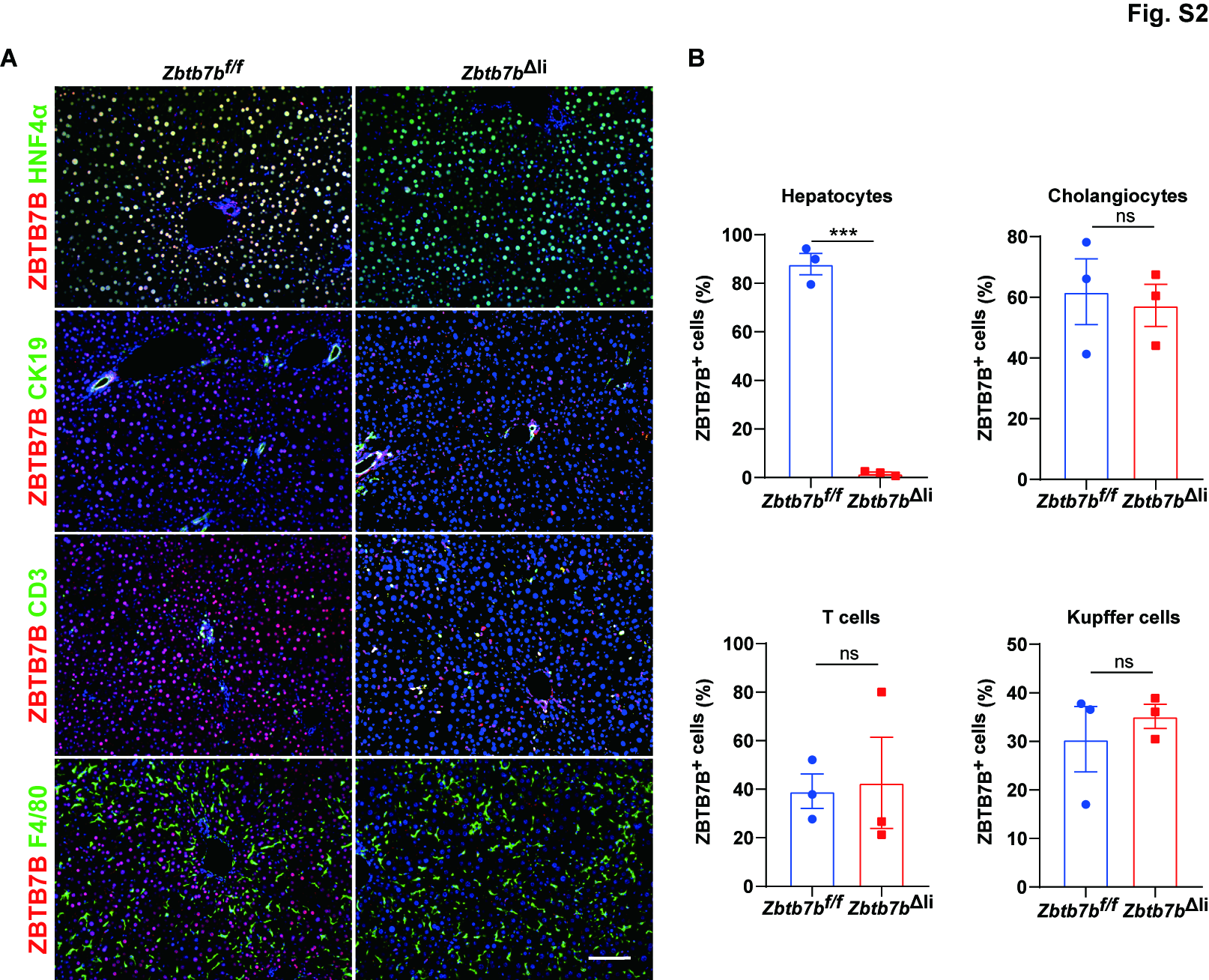
**

**Fig. S2 ZBTB7B is mainly expressed in hepatocytes.**

(A) Multiplex immunostaining of ZBTB7B with HNF4α, cytokeratin 19 (CK19), CD3 and F4/80 on liver sections of *Zbtb7b^f/f^* and *Alb*-*Cre*, *Zbtb7b^f/f^* (*Zbtb7b*^Δli^) adult mice. Magnification: 20×. Scale bars: 100μm.

(B) Quantification of percentage of ZBTB7B-positive cells in hepatocytes, cholangiocytes, T cells and macrophages. n = 3

Data are presented as mean ± SEM. Statistical analyses were performed with two-tailed unpaired student’s *t* test. ****P* < 0.001. ns: Not significant.

**
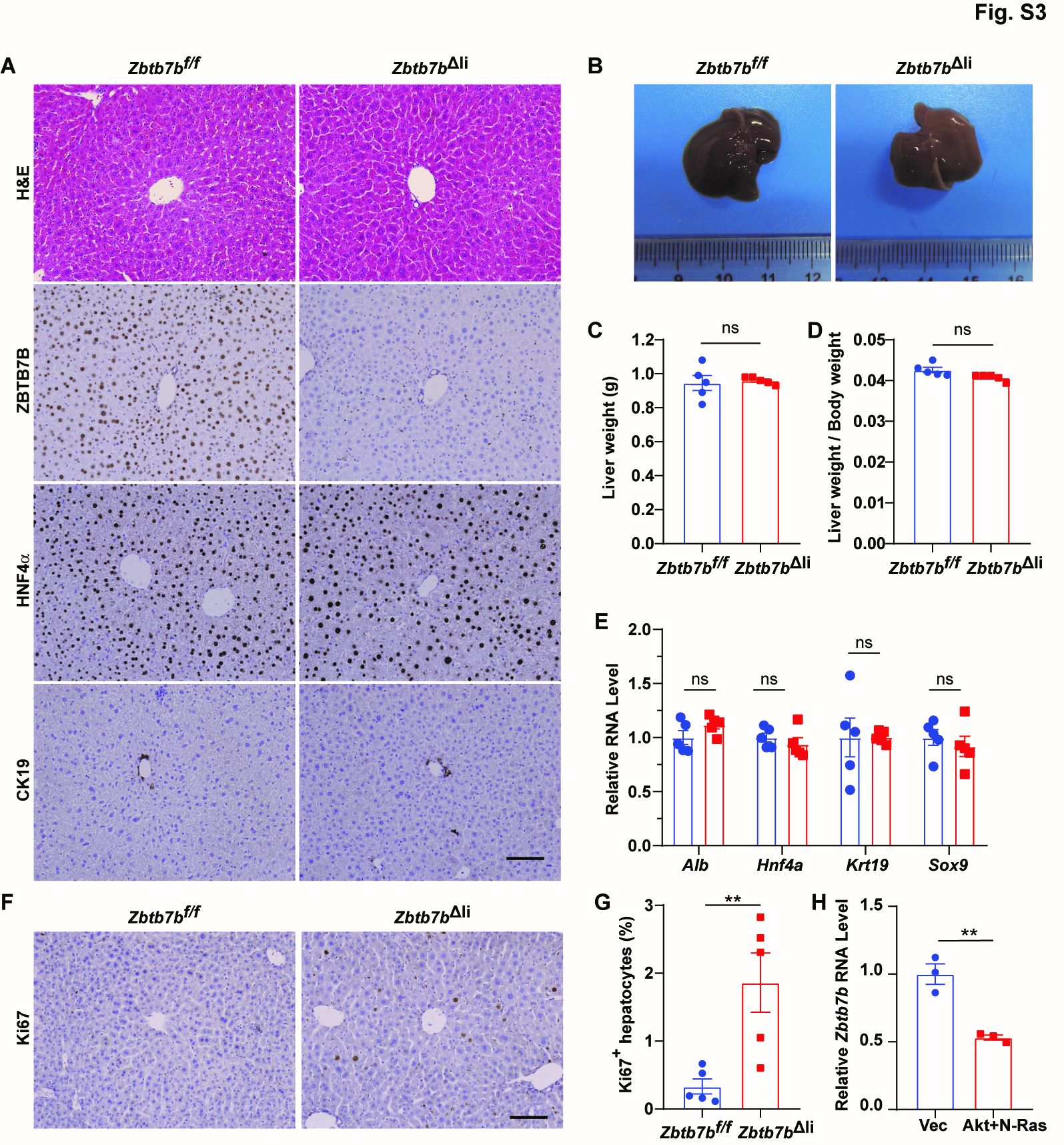
**

**Fig. S3 Ablation of ZBTB7B in hepatocytes does not affect liver development.**

(A) H&E staining and immunohistochemistry of ZBTB7B, HNF4α, cytokeratin 19 (CK19) on liver sections of *Zbtb7b^f/f^* and *Alb*-*Cre*, *Zbtb7b^f/f^* (*Zbtb7b*^Δli^) adult mice. Magnification: 20×. Scale bars: 100μm.

(B) Gross liver images of *Zbtb7b^f/f^* and *Zbtb7b*^Δli^ adult mice.

(C and D) Liver weight (C) and liver/body weight ratio (E) of *Zbtb7b^f/f^* and *Zbtb7b*^Δli^ mice. n = 5

(E) Quantitative RT-PCR analysis of hepatic genes in *Zbtb7b^f/f^* and *Zbtb7b*^Δli^ livers. n = 5

(F and G) Immunohistochemistry of Ki67 on liver section of *Zbtb7b^f/f^* and *Zbtb7b*^Δli^ mice. Magnification: 20×. Scale bars: 100μm. (G) Quantification of (F). n = 5

(H) Quantitative RT-PCR analysis of *Zbtb7b* mRNA levels in livers upon Akt/N-Ras oncogene expression. n = 3

Data are presented as mean ± SEM. Statistical analyses were performed with two-tailed unpaired student’s *t* test. ***P* < 0.01. ns: Not significant.

**
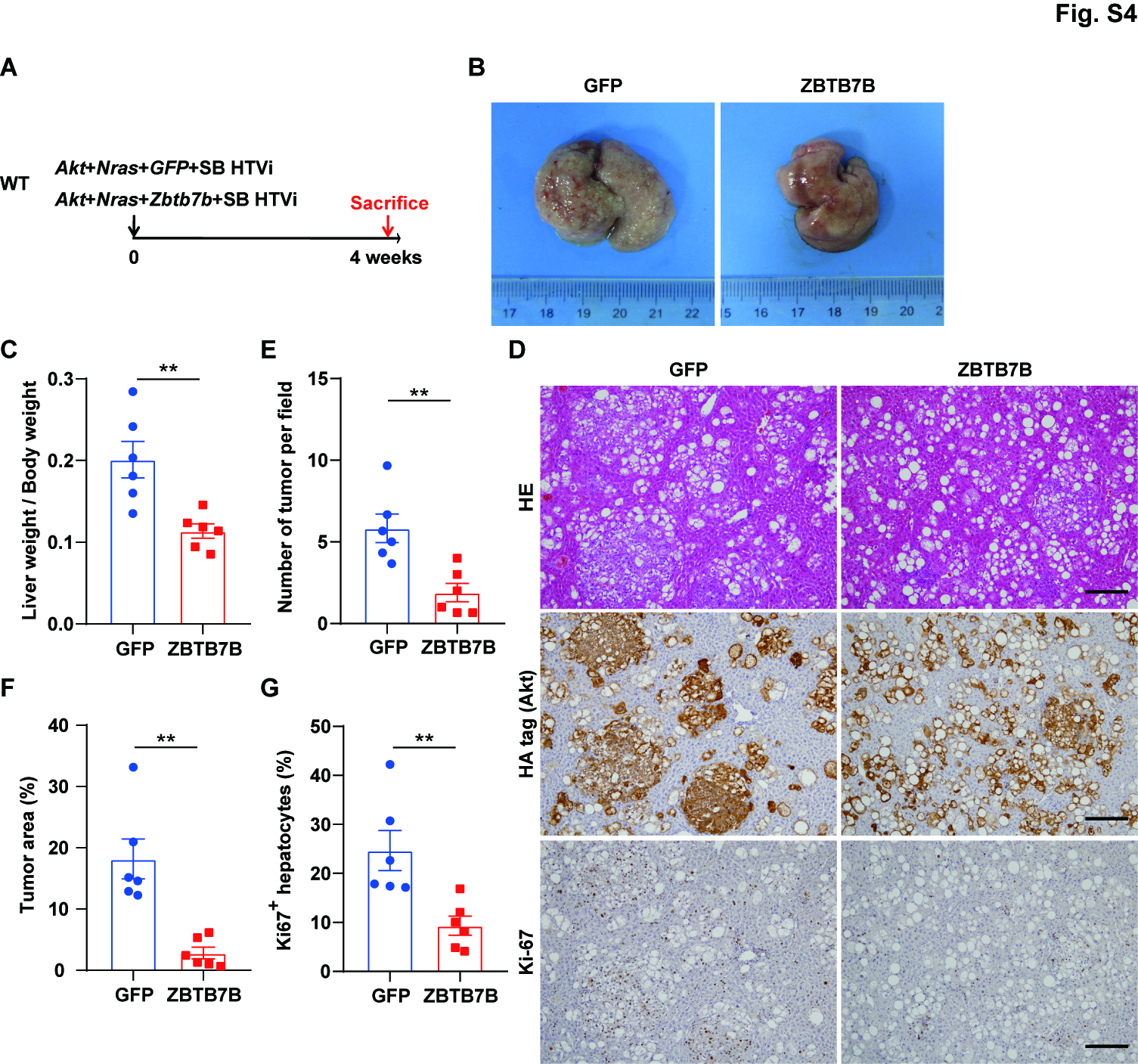
**

**Fig. S4 ZBTB7B delays Akt/N-Ras-induced liver cancer.**

(A) Study design. Hydrodynamic tail vein injection (HTVi) of Akt and N-Ras oncogenes with GFP or ZBTB7B to induce tumor development in wild-type mice. n = 6

(B) Gross liver images.

(C) Liver/body weight ratio 4 weeks after Akt/N-Ras injection.

(D) H&E staining and immunohistochemistry of HA-tag and Ki67 on liver sections. Magnification: 10×. Scale bars: 200μm.

(E and F) Numbers of tumors (E) and percentage of tumor area (F) in livers 4 weeks after Akt/N-Ras injection. n = 6

(G) Percentage of Ki67^+^ hepatocytes in livers 4 weeks after Akt/N-Ras injection. n = 6

Data are presented as mean ± SEM. Statistical analyses were performed with two-tailed unpaired student’s *t* test. ***P* < 0.01.

**
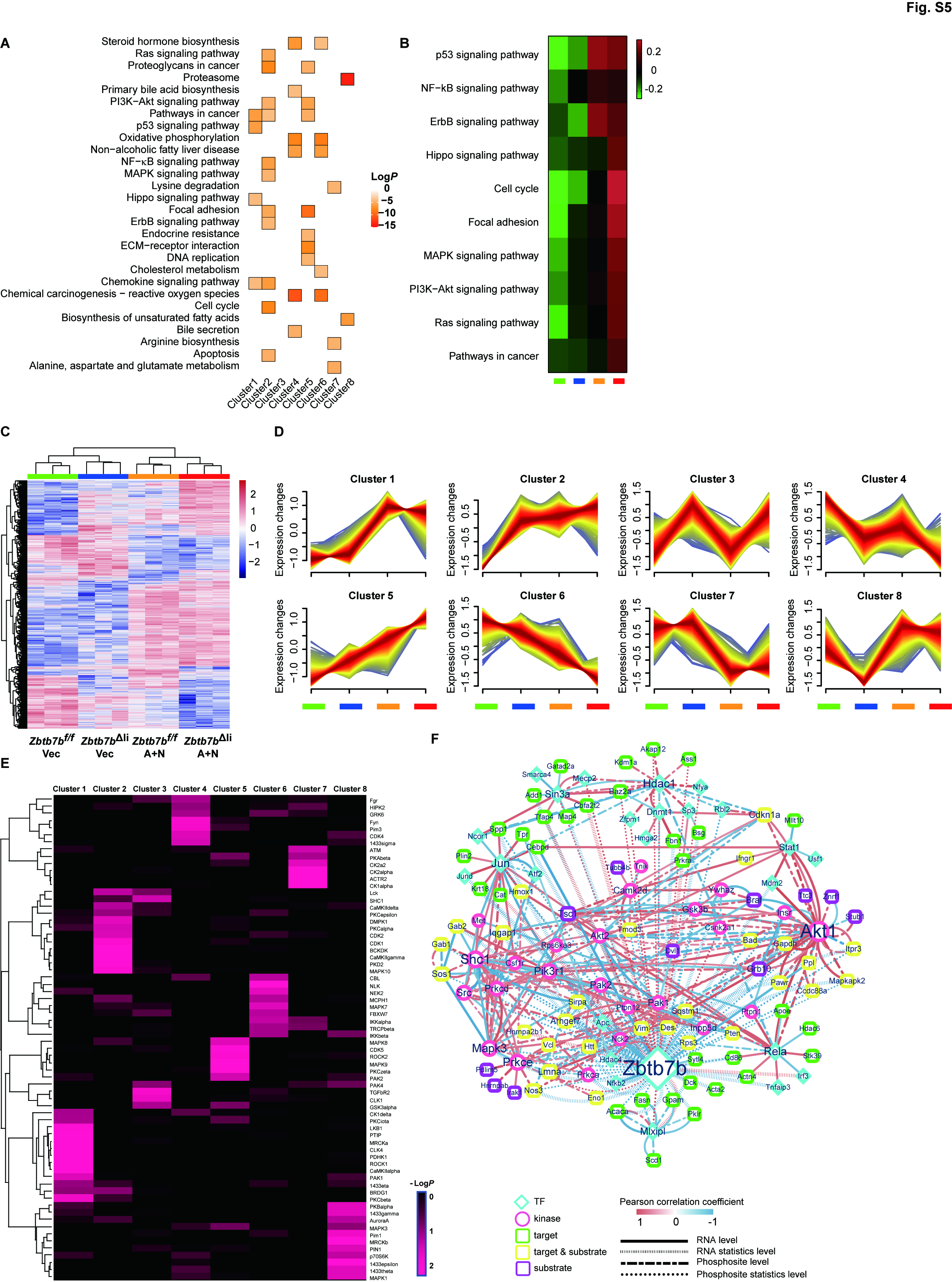
**

**Fig. S5 Multi-omics analyses of ZBTB7B-deficient liver cancer initiation.**

(A) KEGG analysis of differentially expressed genes in each cluster.

(B) Gene set variation analysis (GSVA) scores of signaling pathways in the livers of *Zbtb7b^f/f^* and *Alb*-*Cre*, *Zbtb7b^f/f^* (*Zbtb7b*^Δli^) mice 2 weeks after Akt/N-Ras injection.

(C) Hierarchical clustering analysis for differentially expressed phosphosites (DEPs).

(D) Temporal expression profiling of the DEPs using Mfuzz.

(E) NetworKIN analysis enriched activities of 68 kinases from DEPs.

(F) Protein-protein interaction network regulated by ZBTB7B. The shape and color of nodes represent the functional annotation of the gene. Mint blue diamonds: transcription factors, pink ellipses: kinases, dark green round rectangles: targets, purple round rectangles: substrates, yellow round rectangle: targets and substrates. The edges represent the Pearson correlation coefficient calculated based on gene expression. The line type of edges indicated the data type. Solid line: RNA level, dash dot line: phosphosite level. The color and width of edges indicate strength of correlation. Blue to red: correlation coefficient from -1 to 1. Node size: vitality of the gene.


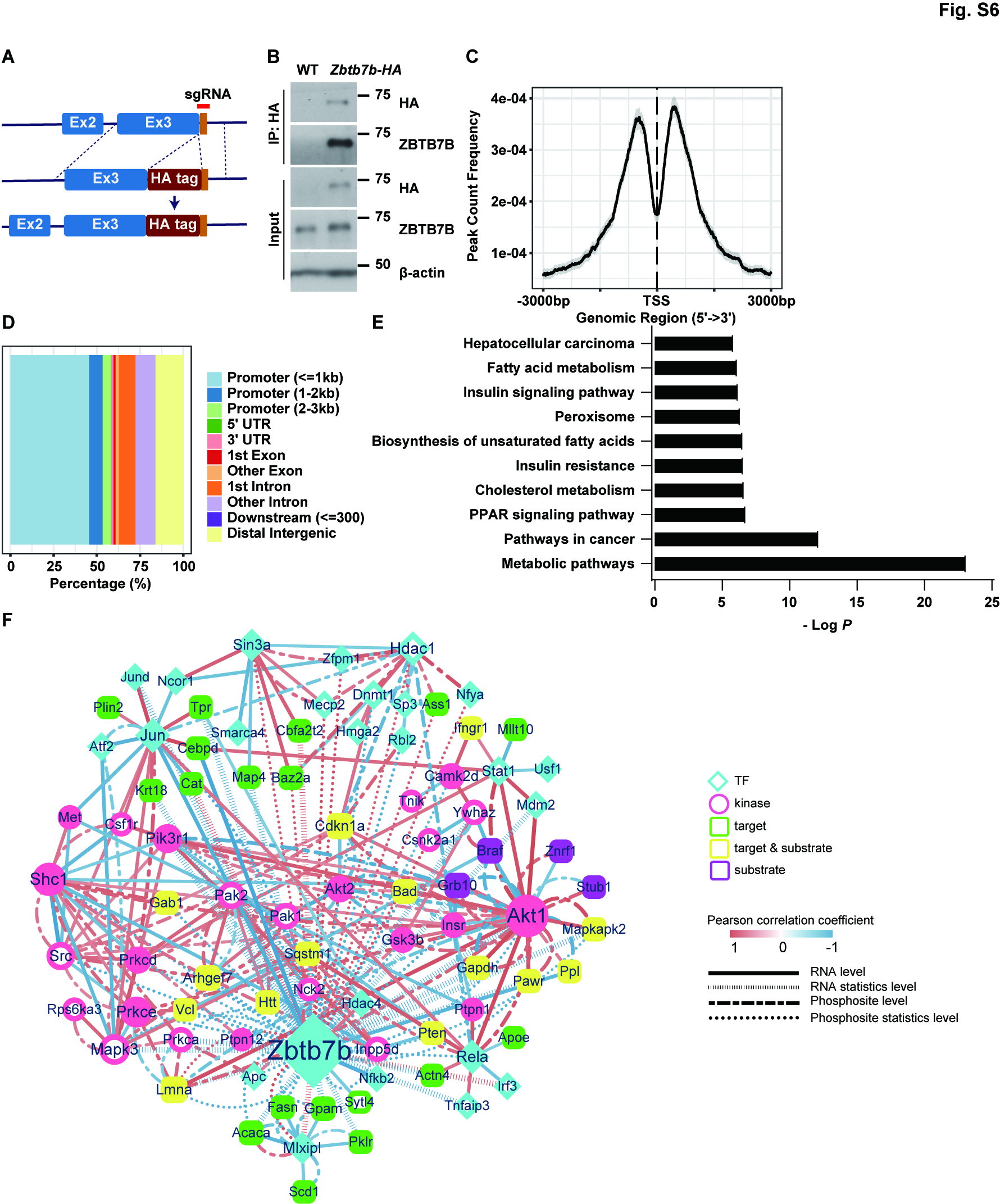


**Fig. S6 ChIP-seq analysis of ZBTB7B genomic occupancy in livers.**

(A) Schematic of *Zbtb7b*-*HA* knock-in strategy with an HA-tag knocked-in in frame before the stop codon of *Zbtb7b* locus.

(B) Anti-HA immunoprecipitants from formaldehyde-crosslinked wild-type and *Zbtb7b*-*HA* liver lysate are detected by anti-HA or ZBTB7B antibodies.

(C-E) Lysates from *Zbtb7b*-*HA* livers were subjected to chromatin immunoprecipitation (ChIP)-sequencing with anti-HA antibody.

(C) Genome distribution of ZBTB7B-binding sites relative to the transcription start site (TSS).

(D) Bar chart of genomic feature of ZBTB7B-binding sites.

(E) Functional enrichment of ZBTB7B-binding sites.

(F) Simplified protein-protein interaction network regulated by ZBTB7B. The shape and color of nodes represent the functional annotation of the gene. Mint blue diamonds: transcription factors, pink ellipses: kinases, dark green round rectangles: targets, purple round rectangles: substrates, yellow round rectangle: targets and substrates. Closed nodes represent direct targets of ZBTB7B. The edges represent the Pearson correlation coefficient calculated based on gene expression. The line type of edges indicated the data type. Solid line: RNA level, dash dot line: phosphosite level. The color and width of edges indicate strength of correlation. Blue to red: correlation coefficient from -1 to 1. Node size: vitality of the gene.


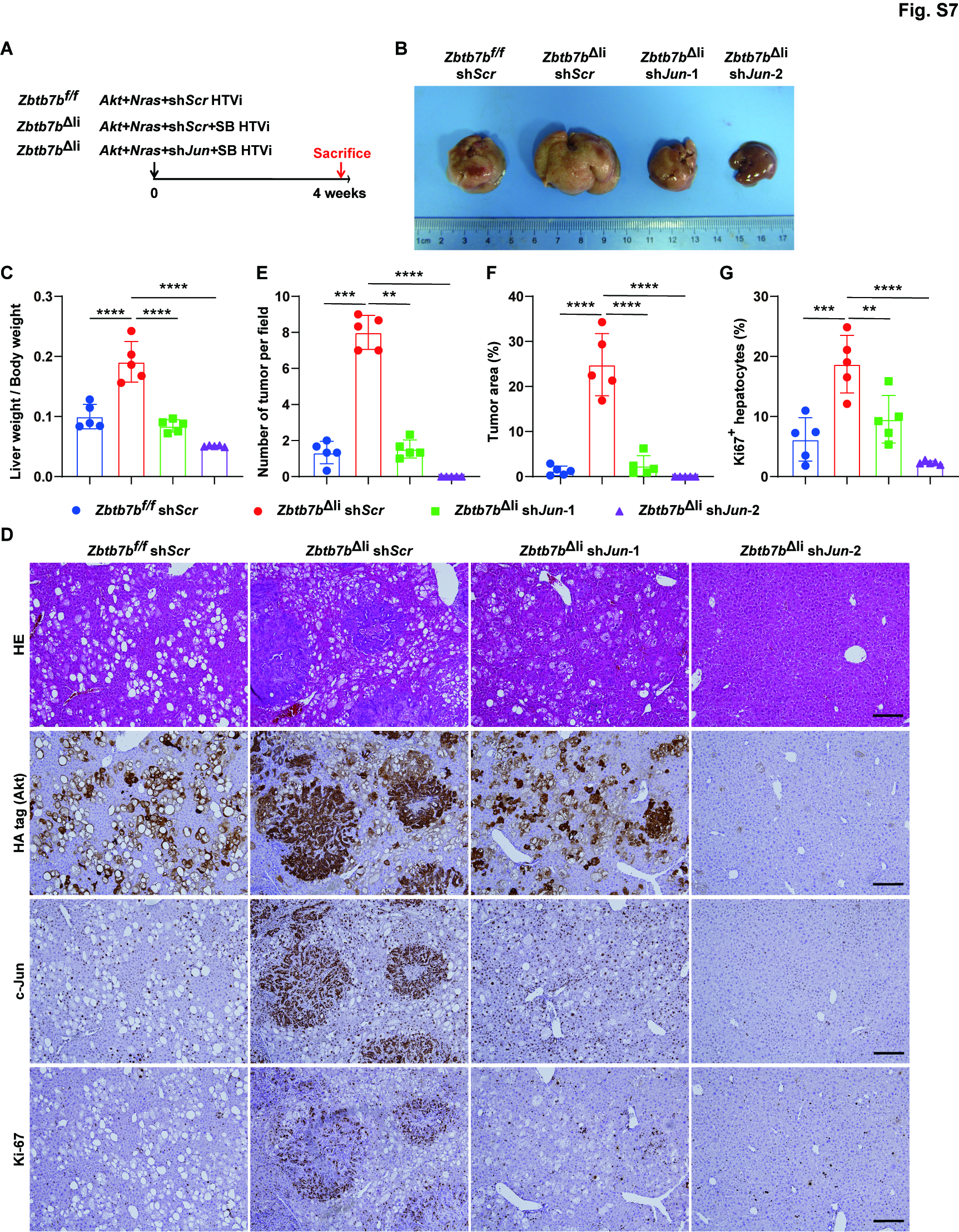


**Fig. S7 c-Jun is essential to accelerated liver cancer development in ZBTB7B-deficient livers.**

(A) Study design. Hydrodynamic tail vein injection (HTVi) of pT3-U6-sh*Scr*-*myrAkt-HA* or pT3-U6-sh*Jun*-*myrAkt-HA* with pT3-*Nras V12* to induce tumor development in *Zbtb7b^f/f^* or *Alb*-*Cre*, *Zbtb7b^f/f^* (*Zbtb7b*^Δli^) mice. Mice were sacrificed 4 weeks after oncogene injection. n = 5

(B) Gross liver images.

(C) Liver/body weight ratio.

(D) H&E staining and immunohistochemistry of HA-tag, c-Jun and Ki67 on liver sections. Magnification: 10×. Scale bars: 200μm.

(E and F) Numbers of tumors (E) and percentage of tumor area (F).

(G) Percentage of Ki67^+^ hepatocytes/tumor cells.

Data are presented as mean ± SEM. Statistical analyses were performed with two-way ANOVA followed by Šídák's multiple comparison test. ***P* < 0.01, ****P* < 0.001, *****P* < 0.0001.

**
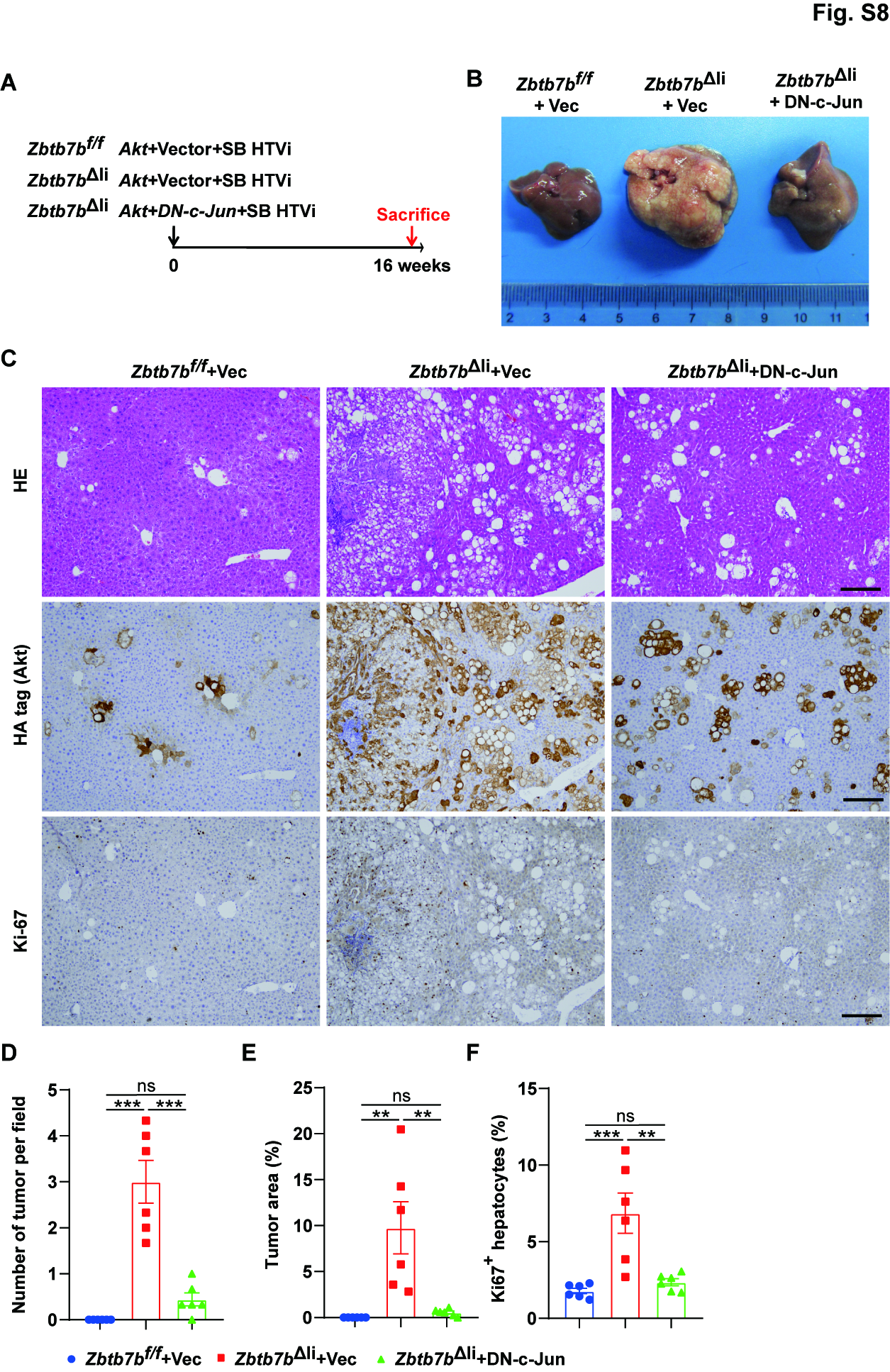
**

**Fig. S8 c-Jun is essential to single oncogene-induced liver cancer development in ZBTB7B-deficient livers.**

(A) Study design. Hydrodynamic tail vein injection (HTVi) of Akt to induce tumor development in *Zbtb7b^f/f^* or *Alb*-*Cre*, *Zbtb7b^f/f^* (*Zbtb7b*^Δli^) mice with pT3-N-FLAG vector control (Vec) or DN-c-Jun. Mice were sacrificed 16 weeks after oncogene injection. n = 6

(B) Gross liver images of *Zbtb7b^f/f^* and *Zbtb7b*^Δli^ mice.

(C) H&E staining and immunohistochemistry of HA-tag and Ki67 on liver sections. Magnification: 10×. Scale bars: 200μm.

(D and E) Numbers of tumors (D) and percentage of tumor area (E).

(F) Percentage of HA tag-positive area (F) and Ki67^+^ hepatocytes (G).

Data are presented as mean ± SEM. Statistical analyses were performed with one-way ANOVA followed by Tukey's multiple comparison test. ***P* < 0.01, ****P* < 0.001. ns: Not significant.


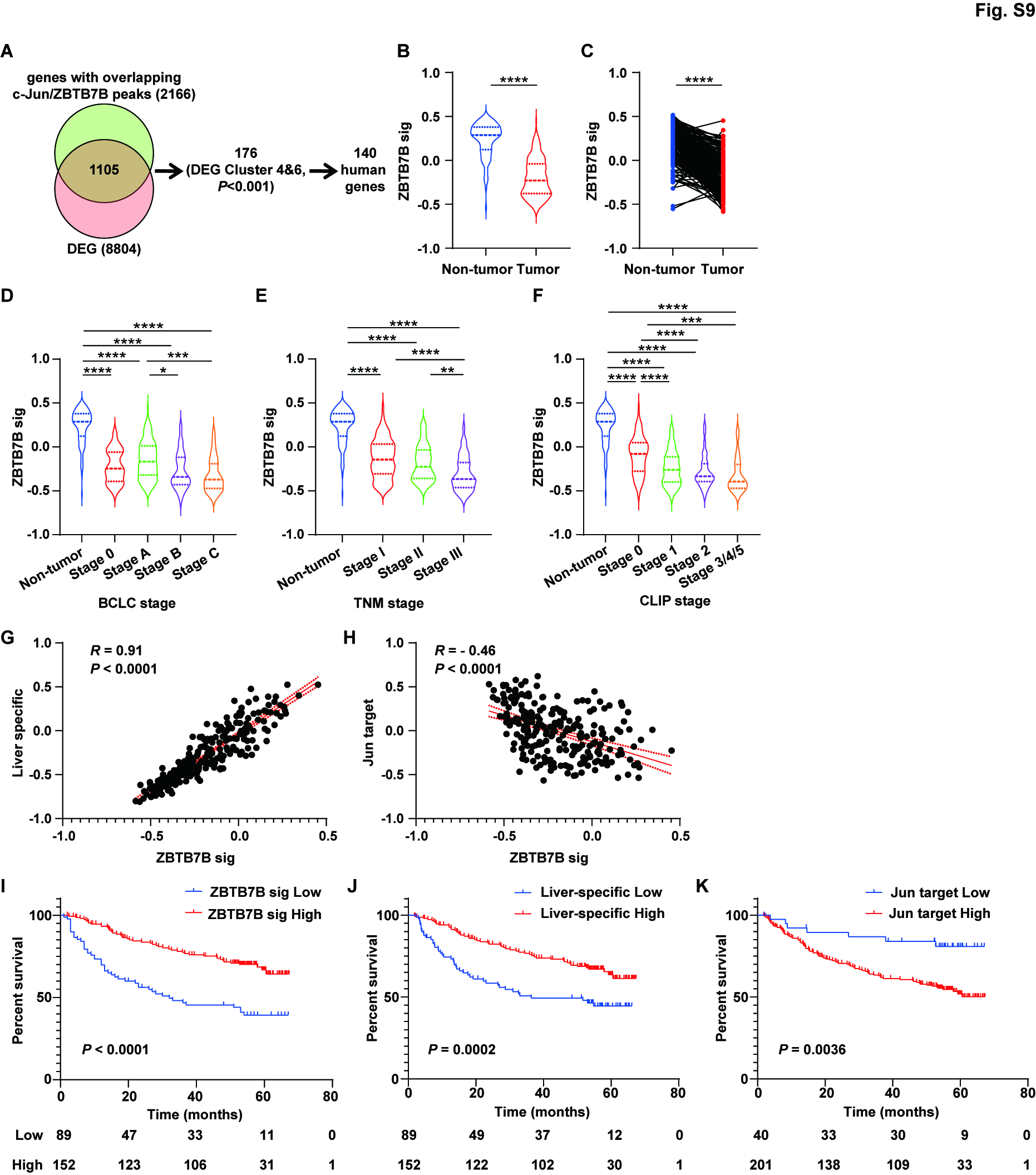


**Fig. S9 ZBTB7B signature predicts liver cancer prognosis.**

(A) Venn diagram depicting overlap of genes with overlapping ZBTB7B and c-Jun binding and DEGs cluster 4 and 6 (*P*<0.001). 140 human orthologous genes are designated as ZBTB7B signature.

Gene Set Variation Analysis (GSVA) scores of ZBTB7B signature, liver-specific genes (HSIAO_LIVER_SPECIFIC_GENES) and c-Jun target genes (MATTHEWS_AP1_TARGETS) were calculated for each HCC patient samples in the GSE14520 cohort.

(B) Comparison of ZBTB7B signature in tumors and adjacent normal tissues.

(C) Comparison of ZBTB7B signature in paired tumors and adjacent normal tissues.

(D-F) Comparison of ZBTB7B signature in HCC tumors of different stages.

(G and H) Pearson correlation of ZBTB7B signature to liver-specific gene (G) and c-Jun target gene (H) expression levels.

(I-K) Probabilities of overall survival of HCC patients according to the expression level of the ZBTB7B signature (I), liver-specific genes (J) and c-Jun target genes (K).

Statistical analyses were performed with two-tailed unpaired (B) or paired (C) student’s *t* test, one-way ANOVA followed by Tukey's multiple comparison test (D-F) or Log-Rank test (I-K). **P* < 0.05, ***P* < 0.01, ****P* < 0.001, *****P* < 0.0001.

**Supplementary tables**

**Table S1.** 8,804 differentially expressed genes (DEGs) and their clustering. The *P*-value was calculated by ANOVA test and *P*-value < 0.05 is considered significant.

**Table S2.** Enriched transcription factors from 8 clusters by TRRUST based on DEGs and DEPs (Differentially expressed phosphosites). The *P*-value was performed by hypergeometric test and *P*-value < 0.001 is considered significant.

**Table S3.** 8,459 differentially expressed phosphosites (DEPs) and their clustering. The *P*-value was calculated by ANOVA test and *P*-value < 0.05 is considered significant.

**Table S4.** Enriched kinases from 8 clusters by NetworKIN based on DEPs. The *P*-value was performed by hypergeometric test and *P*-value < 0.05 is considered significant.

**Table S5.** Protein-protein interaction networks of functional relationship pairs based on DEGs and DEPs. The *P*-value and correlation coefficient were calculated by Pearson correlation test. *P*-value < 0.05 is considered significantly related.

**Table S6.** ZBTB7B ChIP-seq in the *Zbtb7b-HA* knock-in mice.

**Table S7.** c-Jun ChIP-seq in the *Zbtb7b^f/f^* liver.

**Table S8.** c-Jun ChIP-seq in the *Zbtb7b*^Δli^ liver.

**Table S9.** Summary of ZBTB7B signature.

**Table S10.** List of antibodies.

**Table S11.** List of primers.

**Supplementary Materials and methods**

**Mice**

*Alb-cre* mice in 129 background were gift from Dr. Lijian Hui (Center for Excellence in Molecular Cell Science, Chinese Academy of Sciences). *Alb*-*cre* mice were backcrossed to the C57Bl/6 background for >7 generations. *Zbtb7b^f/f^* mice (ref. 1) were gift from Dr. Lie Wang (Zhejiang University). *Zbtb7b^f/f^* mice were crossed with *Alb*-*cre* mice to generate *Alb*-*Cre*, *Zbtb7b^f/f^* (*Zbtb7b*^Δli^) mice. *Zbtb7b*-*HA* knockin mice were generated as described (ref. 2) by Genome Tagging Project (GTP) Center (Center for Excellence in Molecular Cell Science, Chinese Academy of Sciences). sgRNAs of C-terminal of *Zbtb7b* (CATGGAGTCCTCTTAAAAGA) were ligated to the pX330-*mCherry* plasmid (Addgene, #98750) to generate CRISPR-Cas9 plasmid for HA tag Knockin (KI). For the construction of HA tag KI DNA donor, the sequences encoding left homologous arm, HA tag and right homologous arm were amplified and ligated to the linear pMD19T vector with 20 bp overlap in order by Seamless Cloning Kit (Beyotime, D7010S). DKO-AG-haESCs were maintained in DMEM (Millipore) with 15% FBS (Gibco), penicillin/streptomycin, non-essential amino acids, L-glutamine, nucleosides, 2-mercaptoethanol, 1,000 U/mL LIF, 1 μM PD03259010 (Selleck) and 3 μM CHIR99021 (Selleck). DKO-AG-haESCs were transfected with CRISPR-Cas9 plasmid and HA tag KI DNA donor using Lipofectamine 2000 (Life Technologies). 24 hr after transfection, the mCherry-positive haploid cells were enriched by fluorescence-activated cell sorting (FACS, BD AriaII). Single colonies were picked up and positive colonies of HA tag precise KI were selected by genomic DNA PCR amplification for Sanger sequencing. To generate semi-cloned (SC) embryos, DKO-AG-haESCs of *Zbtb7b*-*HA* arrested at M phase by culturing in medium containing 0.05 μg/ml demecolcine for 10 hr were used for intracytoplasmic injection. ICAHCI embryos were cultured in KSOM medium for 24 hr to reach the two-cell stage. 15-20 two-cell embryos were transferred into each oviduct of pseudo-pregnant ICR females at 0.5 days postcoitum (dpc). *Zbtb7b*-*HA* SC mice (F0) were crossed to WT C57BL/6J male mice to generate heterozygotes *Zbtb7b*-*HA* F1 male mice. All mice were housed in a specific pathogen-free environment at the Shanghai Institute of Biochemistry and Cell Biology (SIBCB) and treated in strict accordance with protocols approved by the Institutional Animal Care and Use Committee of SIBCB (Approval number: SIBCB-NAF-15-003-S325-006).

**Plasmids**

pT3-*myr-Akt1-HA*, pT3-*N-Ras V12*, pT3-N-*myc*-*copGFP*, and sleeping beauty transposase (SB) expression plasmids were generously provided by Dr. Xin Chen (UCSF). The coding sequences of *Zbtb7b* and *Jun* were amplified using mouse liver cDNA as template and cloned into pT3-N-myc-copGFP. pT3-N-FLAG vector was generated by replacing the Myc-tag and GFP sequence of pT3-N-myc-copGFP with a FLAG-tag sequence. The dominant negative c-Jun sequence corresponding to amino acid 256-334 was amplified and cloned in frame into pT3-N-FLAG vector. pT3-FLAG-*c-Jun* S63D/S73D was constructed by cloning the c-Jun sequence with mutations of Serine 63 and Serine 73 to aspartic acid in frame into pT3-N-FLAG vector. U6-sh*Scramble* and U6-sh*Jun* fragments were amplified from pLKO.1-U6-sh*Scramble* or pLKO.1-U6-sh*Jun* and inserted into ClaⅠ digested pT3-*myr-Akt1-HA* plasmid through homologous recombination using ClonExpress Ultra One Step Cloning Kit (#C115, Vazyme) to construct pT3-U6-sh*Scramble*-*myr-Akt1-HA* and pT3-U6-sh*Jun*-*myr-Akt1-HA*. The target sequences are CAACAAGATGAAGAGCACCAA (sh*Scramble*), GCAAAGATGGAAACGACCTTC (sh*Jun*-1) and GGAACAGGTGGCACAGCTTAA (sh*Jun*-2).

**Hydrodynamic injection**

Hydrodynamic injection was performed as previously described (ref. 3, 4). Briefly, 10μg of pT3-*myr-Akt1-HA* and pT3-*N-Ras V12* along with sleeping beauty transposase (SB) were diluted in 2 mL sterile PBS. The plasmid solution was injected into the lateral tail vein of 6-week-old male mice in 5 to 7 seconds. For Akt-induced liver cancer, 20 μg of pT3-*myr-Akt1-HA* together with SB in 2 mL PBS were injected through mice tail vein. 10 μg pT3-*Zbtb7b*, pT3-*DN-Jun* or pT3-*Jun* S63D/S73D was applied for hydrodynamic injection. Total pT3 plasmids and SB plasmid were in a ratio of 25:1.

**Histology and immunohistochemistry**

Mice were anaesthetized and livers were isolated and fixed in 4% PFA followed by embedding in paraffin. Paraffin-embedded tissues were sectioned and stained with hematoxylin and eosin (H&E). Pictures were taken at 3 random fields at 4×, 10×, 20× or 40× magnifications. The amount and area of tumors were quantified using ImageJ (US National Institute of Health). The immunohistochemical staining were performed as previously described (ref. 5). Briefly, deparaffined and rehydrated 5 μm liver sections were subjected to heat-induced epitope retrieval with 10mM Citrate at pH 6.0 or 10mM EDTA/1mM Tris at pH 9.0. Sections were incubated with diluted primary antibodies at 4 °C overnight, followed by incubation with biotinylated secondary antibodies and horseradish peroxidase-conjugated ABC complex. The primary antibodies used are listed in Supplementary Table S10. Signals were developed with DAB chromogen and counterstained with hematoxylin. Sections were viewed under microscope BX53 with an UPlanSAPO ×20 objective/0.75 (OLYMPUS, Inc.). Images were captured with a digital camera (DP71; OLYMPUS, Inc.) and Digital Acquire software (DPController; OLYMPUS, Inc.). Multiplex immunofluorescent staining was performed using Opal^TM^ fluorophores (PerkinElmer). The H-scores and percentage of ZBTB7B-positive cells in HNF4α, CK19, CD31 or F4/80-positive cells are analyzed were quantified using InForm® software (PerkinElmer).

**Western blot and quantitative RT-PCR analysis**

Livers were homogenated in SDS sample buffer or Trizol reagents (Invitrogen). Western blot and quantitative RT-PCR analyses were performed as previously described (ref. 5). The primary antibodies and primers used are listed in Supplementary Table S10 and S11, respectively. Gene expression levels were normalized to *Actin*. All experiments were performed at least twice.

**RNA-seq analysis**

Total RNA was extracted and purified from livers using TRIZOL (Invitrogen). Three biological replicates were subjected to complementary DNA library preparation according to the Illumina standard protocol. Libraries were sequenced on the NovaSeq 6000 with 150bp paired-end sequencing (Berry Genomics). After cut adapters by Trimgalore (v.0.5.0), RNA-seq data was mapped to mouse mm10 reference genome by STAR (v.2.9). FeatureCount in Subread package (v.1.6.4) was used to counting reads. R package DESeq2 (v.1.24.0) was used to do normalization and differential expression analysis. GSVA was run on default parameters using R package GSVA (v.1.32.0) (ref. 6). Gene ontology analysis was performed using g:profiler (ref. 7). GSEA was performed on GSEA application (v.4.0.3) with gene signatures obtained from the MSigDB database version 7.5.1. Statistical significance was assessed by comparing the enrichment score to enrichment results generated from 1,000 random permutations of the gene set to obtain *P*-value (nominal *P*-value) (ref. 8).

**ChIP-seq analysis**

Briefly, livers were perfused with PBS for 1 minute to remove blood cells. 100 mg perfused liver tissues were cut into small pieces, cross-linked with 1.5% formaldehyde for 10 min at room temperature, then washed with cold PBS for three times. Tissues were homogenized by dounce homogenizer in cold PBS and cells were collected by centrifugation. For ZBTB7B ChIP, chromatin was sonicated to an average size of 200-500 bp in RIPA buffer (0.1% SDS, 1% Triton X-100, 10mM Tris-HCl (pH 7.4), 1mM EDTA (pH 8.0), 0.1% sodium deoxycholate) by Ultrasonic Homogenizer (SCIENTZ). 10μg sonicated chromatin was incubated with 5 μl HA antibody (#3724S, Cell Signaling) in RIPA buffer with 0.3 M NaCl at 4 °C overnight. c-Jun ChIP was performed with SimpleChIP Plus Kit (#9005, Cell Signaling). Briefly, cross-linked chromatin was digested to an average size of 200–500 bp with micrococcal nuclease. 10 μg sonicated chromatin was incubated with 5 μl c-Jun antibody (#9165, Cell Signaling) or 1 μl normal rabbit IgG (#2729, Cell Signaling) at 4 °C overnight. Protein A Dynabeads (#10002D, Invitrogen) were added to the ChIP reactions and incubated for 3 additional hours at 4 °C to collect the immunoprecipitated chromatin. Subsequently, Dynabeads were washed twice with 1 mL of RIPA buffer with 0.3 M NaCl, twice with 1 mL of High Salt buffer (50 mM HEPES (pH7.5), 1 mM EDTA (pH8.0), 0.5 M NaCl, 1% Triton X-100, 0.1% sodium deoxycholate), twice with 1 mL of LiCl buffer (10 mM Tris-HCl, 1 mM EDTA, 0.5% sodium deoxycholate, 0.5% NP-40, 250 mM LiCl, pH 8.0), and twice with 1 mL of TE buffer (10 mM Tris-HCl, 1 mM EDTA, pH 8.0). The chromatin was eluted in SDS elution buffer (1% SDS, 10 mM EDTA, 50 mM Tris-HCl, pH 8.0) followed by reverse crosslinking at 65°C overnight. ChIP DNA were treated with RNaseA (5 μg/mL) and protease K (0.2 mg/mL), and purified by phenol/chloroform extraction. The concentration of DNA was determined by Qubit (#EQ121-01, Vazyme). The eluted DNA was subjected to library preparation using library preparation kit (#ND607, Vazyme) according to the manufacturer’s instructions. The libraries were sequenced on Illumina Novaseq. After cut adapters by fastp (v.0.20.1), ChIP-seq data was mapped to mouse mm10 reference genome by Bowtie2 (v.2.3.1). Peak detection was performed using the MACS2 (v.2.1.1). The BAM files were transformed to CPM-normalized Bigwig files by Deeptools (v.3.5.0). The heatmaps and enrichment profiles were visualized with plotHeatmap and plotProfile function in Deeptools, respectively. MA plot was generated by MAnorm (v.1.3.0) (ref. 9). The visualization and annotation were performed by IGV (v.2.11.2) and R package ChIPseeker (v.1.30.3), respectively. Peak overlapping was computed by using the default bedtools intersect intervals functions with at least 1 base pair overlap. c-Jun ChIP-seq signals centered on the summit of ZBTB7B peaks were performed with Deeptools v3.5.0 and visualized with plotProfile function in Deeptools.

**Mass spectrometry analysis**

Mouse livers was frozen at -80℃ and cut into small pieces. Tissues were lysed with SDT lysis buffer for total protein extraction followed by 5 min of heating at 95 °C and 2 min of sonication (10 second on and 5 second off, power 50 Watts). The tissue and cell debris were pelleted by centrifugation at 12,000×*g* for 20 min and the supernatants containing proteins were collected for subsequent analysis. Protein sample was digested by filter-aided sample preparation protocol (FASP) (ref. 10) using 10 kDa centrifugal filter tubes (PALL) in 100 mM NH_4_HCO_3_ solution at 37 ℃. Trypsin (Promega) was added in two rounds. The first round was lasting 12 h with 1:50 of total protein amount, and the second round was lasting additional 4 h with equal trypsin amount. Each peptide mixture was eluted by centrifugation and dried by speed-vac.

The phosphopeptide enrichment was performed using High-Select Fe-NTA kit (Thermo Fisher Scientific) according to the kit manual and previous report (ref. 11) with some following modifications. In brief, after the peptide-resin mixture was incubated for 30 min with thrice gentle blowing at room temperature, transfer them into a homepacked one-layer Empore-C8 StageTip (ref. 12) to remove nonspecific peptides and elute phosphopeptides. Elutes were immediately dried by speed-vac at 45 ℃ for mass spectrometry analysis.

The sample was analyzed on a Thermo Scientific EASY-nLC nanoflow LC. The peptides were resolved using 0.1% formic acid in ddH_2_O and was separated using a home-made micro-tip C18 column (75 mm × 200 mm) packed with ReproSil-Pur C18-AQ, 3.0 mm resin (Dr. Maisch GmbH, Germany). Briefly, the sample was loaded onto a nano-C18 column and separated at a flow rate of 300 nL/min with following 120 min gradients: 0-2 min, 2-8% buffer B; 2-90 min, 8-23% B; 90-105 min, 23-40% B; 105-110 min, 40-100% B; 110-120 min, 100% B. Thermo Scientific Q Exactive HF-X Hybrid Quadrupole-Orbitrap mass spectrometer was carried out running data independent analysis (DIA) mode. Full MS performed 120K resolution MS scan @ m/z 200. The MS AGC target value was set at 3e6 with 50 ms of max injection time. Tandem mass spectra were acquired using a collision energy of 28, resolution of 30K, maximum inject time of 50 ms and AGC target of 1e5. Fragment analysis was subdivided into 42 DIA isolation windows with an equal 20 m/z widths from 380 m/z to 1220 m/z.

**Phosphoproteomic data processing and analysis**

All phosphoproteome DIA runs were analyzed by Spectronaut^TM^ 15 (Biognosys Inc.) according to directDIA workflow with default settings. Reference FASTA files for mouse was downloaded from UniProt on May 2021, combining with the fusion sequence of iRT (Biognosys Inc.). Phosphoproteomic database search services by SpectronautTM software package were provided by Shanghai Applied Protein Technology Co., Ltd. (Shanghai, China). A maximum number of 5 modifications per peptide were allowed for each peptide. Enzyme specificity was set as trypsin/P. The maximum missing cleavage site was set as 2. Carbamidomethyl (C) was set as fixed modifications. Oxidation (M), Acetyl (Protein N-term), and phospho (STY) were set as variable modifications. Calibration was set to non-linear iRT calibration with precision iRT enabled. Identification was performed using 1% q-value cutoff on precursor, peptide, and protein level. Quantity was determined on MS/MS level using area of XIC peaks with enabled cross run normalization. For phosphoproteomic analysis, minor quantified (Peptide) grouping was set by modified sequence and PTM localization was activated and probability cutoff set to 0, in order to summarize phosphopeptide or phosphosite later. To combine Spectronaut precursor quantifications into consensus phosphosites, transformation of the Spectronaut normal report and calculation of stoichiometry values was performed using a reported plugin Peptide Collapse in Perseus (1.6.8.0) (ref. 13). As a result, we present the phosphoproteome included 34,220 quantified phosphosites. Based on phosphor-expression profiles, significant 8459 sites (ANOVA *P*-value < 0.05) as differentially expressed phosphosites (DEPs) were classed into 8 clusters and used to do further kinase-substrate and kinase enrichment analysis.

To estimate changes in a kinase’s activity, kinase-substrate site relationships from NetworKIN 3.0 (ref. 14) with score more than 0.9 were used as the K-S sources. The significantly expressed 8,459 sites were mapped to their upstream kinases and count K-S relationships for all 8 clusters. For the kinase enrichment analysis, the thresholds for significantly enriched kinases were hypergeometric test *P*-value less than 0.05 and substrate number ≥ 3 at least in one cluster. And 68 enriched kinases were selected.

**Gene ontology and functional enrichment analysis**

Functional enrichment analysis was performed for biological processes of Gene Ontology (GO) analysis and Kyoto Encyclopedia of Genes and Genomes (KEGG) pathway analysis via the Metascape (http://www.metascape.org/) online tool. Analyses were carried out with the default enrichment parameters, among which minimum overlap is 3, *P*-value cutoff is 0.01 and minimum enrichment is 1.5. The enrichment results in PaGenBase and TRRUST were given.

**Protein-protein interaction (PPI) network construction**

The PPI networks at RNA level and phosphosite level were arranged separately and then integrated to obtain the total functional PPI network. Intersection (1,373 genes) of differentially expressed genes (8804 genes) and phosphosites (2,533 genes mapped) were selected. Paired relationships were obtained using MIPPIE, TRRUST and additional known relationships with Zbtb7b (UniProt and STRING). Functional PPIs were screened out according to the kinase enrichment analysis and transcription factor enrichment analysis, which included 68 enriched kinases based on DEPs and 65 transcription factors based on DEGs (52 TFs) and DEPs (13 TFs). At least one node of the pairing interaction is a kinase or transcription factor. The above PPIs were uploaded to STRING (http://string-db.org/) for a complementary view of potential interactions. The settings were as follows: the experiments and databases options for active interaction source were selected and their relationships were remained with highest confidence (Score ≥ 0.9). Pearson correlation test was performed to count edge values according to the expression profiles at each omics level, and *P*-value 0.05 was used as the threshold of significance. On this basis, the nodes correlated with Zbtb7b (*P*-value < 0.05) were identified as statistically relevant nodes and were incorporated into the network. PPI networks at phosphosite level might contain redundant upregulated or downregulated edges due to different phosphorylation regulatory sites for the given nodes. Therefore, we took the average of correlation coefficients greater than 0 or less than 0 separately to simplify the network. There were 97 nodes and 145 edges at RNA level, 46 nodes and 45 edges at RNA statistic level, 80 nodes and 123 edges at phosphosite level and 55 nodes and 55 edges at phosphosite statistics level, respectively. All those nodes and edges were incorporated into a network with 122 nodes and 368 edges (Supplementary Table S5, Fig. S5F). In order to show the importance of functional or evidential nodes, and to reduce the complexity of the network, we eliminated nodes that were neither functional genes nor in ChIP-Seq and network regulated by ZBTB7B had 85 nodes and 270 edges (Supplementary Table S5, Fig. S6F). Fig. 4D only contained the subnetwork of DEGs and DEPs synergistically regulated by ZBTB7B and Akt/N-Ras oncogenes (clusters 2, 4, 5 and 6), which remained 26 nodes and 51 edges. Cytoscape (version 3.8.0, http://www.cytoscape.org/) was used to draw network characteristics above.

**Supplementary references**

1. Vacchio MS, Wang L, Bouladoux N, Carpenter AC, Xiong Y, Williams LC, et al. A ThPOK-LRF transcriptional node maintains the integrity and effector potential of post-thymic CD4+ T cells. *Nat Immunol* **15,** 947-956 (2014)

2. Zhong C, Yin Q, Xie Z, Bai M, Dong R, Tang W, et al. CRISPR-Cas9-Mediated Genetic Screening in Mice with Haploid Embryonic Stem Cells Carrying a Guide RNA Library. *Cell Stem Cell* **17,** 221-232 (2015)

3. Liu F, Song Y & Liu D. Hydrodynamics-based transfection in animals by systemic administration of plasmid DNA. *Gene Ther* **6,** 1258-1266 (1999)

4. Ho C, Wang C, Mattu S, Destefanis G, Ladu S, Delogu S, et al. AKT (v-akt murine thymoma viral oncogene homolog 1) and N-Ras (neuroblastoma ras viral oncogene homolog) coactivation in the mouse liver promotes rapid carcinogenesis by way of mTOR (mammalian target of rapamycin complex 1), FOXM1 (forkhead box M1)/SKP2, and c-Myc pathways. *Hepatology* **55,** 833-845 (2012)

5. Wu Y, Cao Y, Xu K, Zhu Y, Qiao Y, Wu Y, et al. Dynamically remodeled hepatic extracellular matrix predicts prognosis of early-stage cirrhosis. *Cell Death Dis* **12,** 163 (2021)

6. Hanzelmann S, Castelo R & Guinney J. GSVA: gene set variation analysis for microarray and RNA-seq data. *BMC Bioinformatics* **14,** 7 (2013)

7. Raudvere U, Kolberg L, Kuzmin I, Arak T, Adler P, Peterson H, et al. g:Profiler: a web server for functional enrichment analysis and conversions of gene lists (2019 update). *Nucleic Acids Res* **47,** W191-W198 (2019)

8. Subramanian A, Tamayo P, Mootha VK, Mukherjee S, Ebert BL, Gillette MA, et al. Gene set enrichment analysis: a knowledge-based approach for interpreting genome-wide expression profiles. *Proc Natl Acad Sci U S A* **102,** 15545-15550 (2005)

9. Shao Z, Zhang Y, Yuan GC, Orkin SH & Waxman DJ. MAnorm: a robust model for quantitative comparison of ChIP-Seq data sets. *Genome Biol* **13,** R16 (2012)

10. Wiśniewski JR, Zougman A, Nagaraj N & Mann M. Universal sample preparation method for proteome analysis. *Nature Methods* **6,** 359-362 (2009)

11. Gao Q, Zhu H, Dong L, Shi W, Chen R, Song Z, et al. Integrated Proteogenomic Characterization of HBV-Related Hepatocellular Carcinoma. *Cell* **179,** 561-577 e522 (2019)

12. Rappsilber J, Mann M & Ishihama Y. Protocol for micro-purification, enrichment, pre-fractionation and storage of peptides for proteomics using StageTips. *Nature Protocols* **2,** 1896-1906 (2007)

13. Bekker-Jensen DB, Bernhardt OM, Hogrebe A, Martinez-Val A, Verbeke L, Gandhi T, et al. Rapid and site-specific deep phosphoproteome profiling by data-independent acquisition without the need for spectral libraries. *Nat Commun* **11,** 787 (2020)

14. Horn H, Schoof EM, Kim J, Robin X, Miller ML, Diella F, et al. KinomeXplorer: an integrated platform for kinome biology studies. *Nature Methods* **11,** 603-604 (2014)
