# Supplementary material for: ZBTB7B is a permissive regulator of hepatocellular carcinoma initiation by repressing c-Jun expression and function
Source: Cell Death Dis. 2024 Jan 15;15(1):55. doi: 10.1038/s41419-024-06441-y (PMC10789742; doi:10.1038/s41419-024-06441-y)
Supplement: Supplementary file 3 — Original Data File [file 41419_2024_6441_MOESM3_ESM.pdf]

# Full unedited gel for Figure 4I

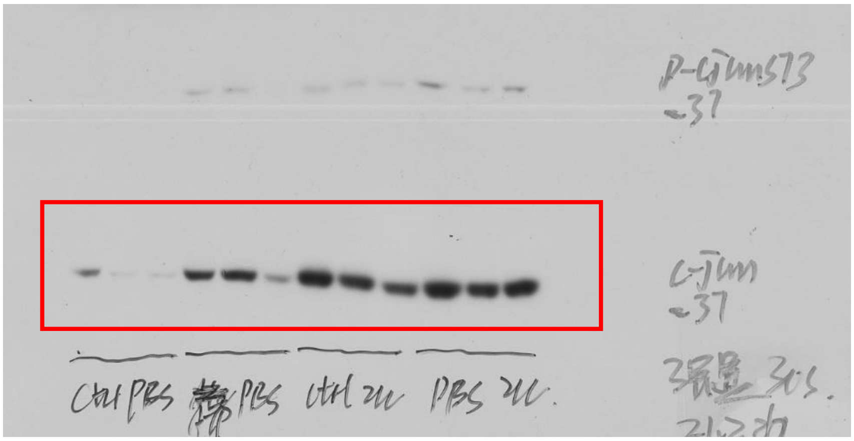


αc-Jun

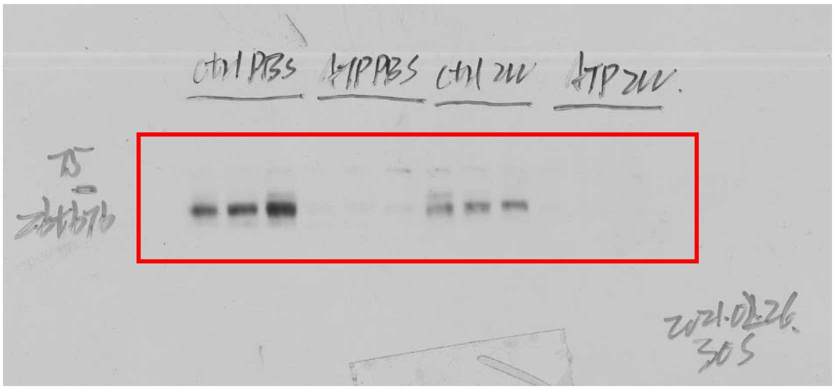


αZBTB7B

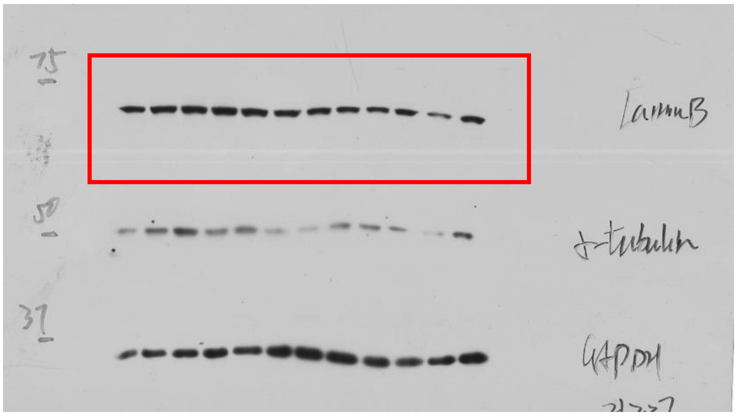


αLamin B1

# Full unedited gel for Figure 4J

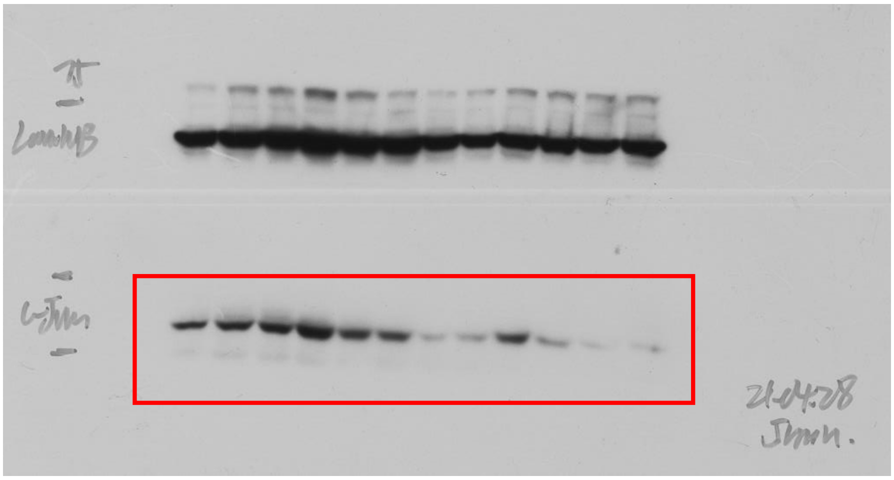

αc-Jun

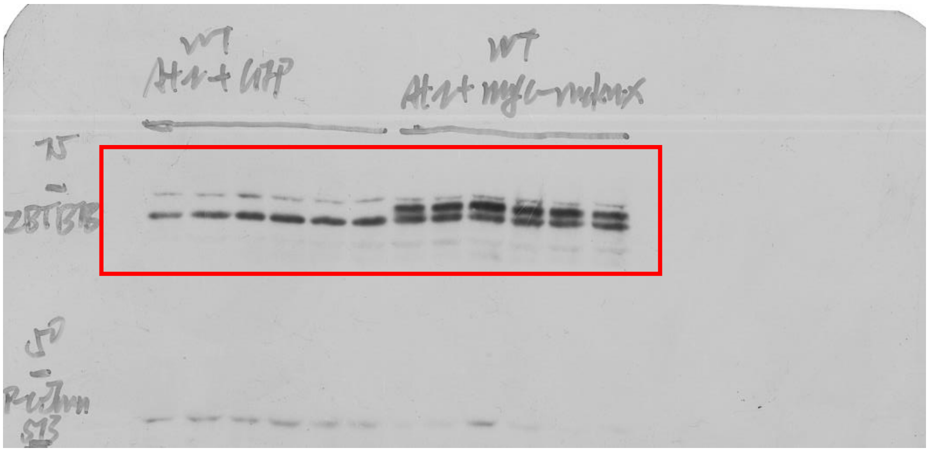

αZBTB7B

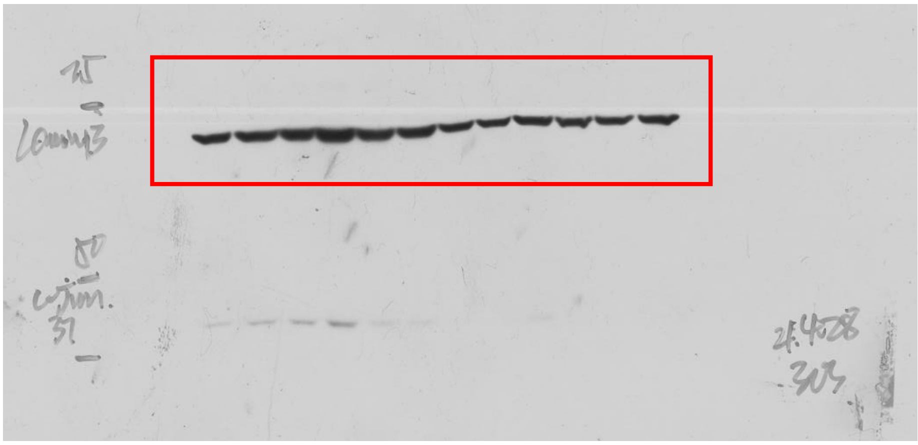

αLamin B1

## Full unedited gel for Figure S1G

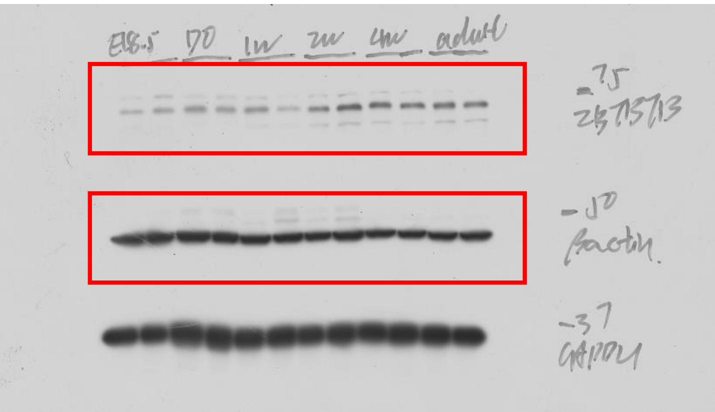


αZBTB7B

αActin

## Full unedited gel for Figure S6B

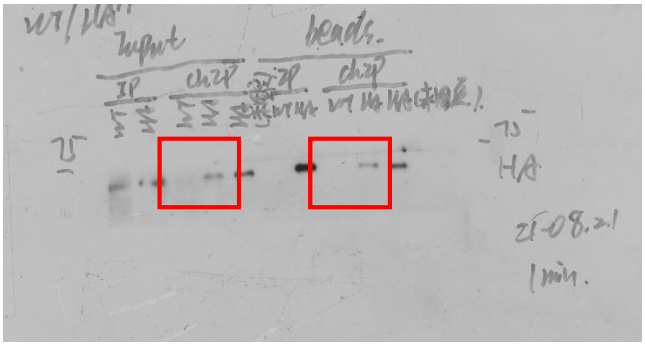


αHA
Left: input
Right: IP

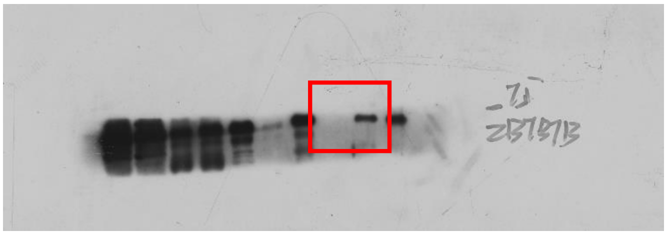


αZBTB7B (IP)

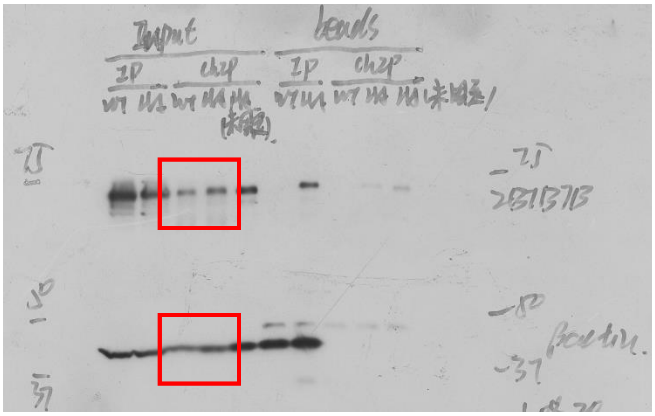


αZBTB7B (Input）

αActin (Input）
